# Operationalising a real-time research ethics approach: supporting ethical mindfulness in agriculture-nutrition-health research in Malawi

**DOI:** 10.1186/s12910-021-00740-1

**Published:** 2022-01-11

**Authors:** Limbanazo Matandika, Kate Millar, Eric Umar, Edward Joy, Joseph Mfutso-Bengo

**Affiliations:** 1 Center for Bioethics in Eastern and Southern Africa, University of Malawi, College of Medicine, Private Bag 360, Blantyre, Malawi; 2 Centre for Applied Bioethics, Schools of Biosciences and Veterinary Medicine and Science, University of Nottingham, Sutton Bonington Campus, Loughborough, LE12 5RD UK; 3 Health Systems and Policy Department, University of Malawi, College of Medicine, Private Bag 360, Blantyre, Malawi; 4 Faculty of Epidemiology and Population Health, London School of Hygiene and Tropical Medicine, Keppel Street, London, WC1E 7HT UK

**Keywords:** Ethical considerations, Ethical mindfulness, Ethics in agriculture-nutrition, Interactive sessions, Participant adherence, Real-time research ethics approach, Responsiveness, Study obligations

## Abstract

**Background:**

There have been notable investments in large multi-partner research programmes across the agriculture-nutrition-health (ANH) nexus. These studies often involve human participants and commonly require research ethics review. These ANH studies are complex and can raise ethical issues that need pre-field work, ethical oversight and also need an embedded process that can identify, characterise and manage ethical issues as the research work develops, as such more embedded and dynamic ethics processes are needed. This work builds on notions of ‘ethics in practice’ by developing an approach to facilitate ethical reflection within large research programmes. This study explores the application of a novel ‘real-time research ethics approach’ (RTREA) and how this can support ethical mindfulness. This involves embedding ethical analysis and decision-making within research implementation, with a continuous dialogue between participants and researchers. The aim is to improve ethical responsiveness and participant experience, which in turn may ethically support adherence and retention. In this case study, a bioethics team (BT) was embedded in a community-based randomised, controlled trial conducted in rural Malawi, titled the ‘Addressing Hidden Hunger with Agronomy’. To identify ethical issues, the researchers conducted ten focus group discussions, fourteen in-depth interviews with key informants, two workshops, observed two sensitisation and three activity meetings conducted by the trial team, and analysed fifteen reports from pre-trial to trial implementation.

**Results:**

The RTREA facilitated the identification of social and ethical concerns and made researchers aware of participants’ ‘lived research experience’. To address concerns and experiences, the BT worked with researchers to facilitate conversation spaces where social and ethical issues were discussed. Conversation spaces were designed to create partnerships and promote participatory methods to capture trial participants’ (TPs) perspectives and experiences.

**Conclusions:**

The use of RTREA showed the value of real-time and continuous engagement between TPs and researchers. These real-time processes could be embedded to complement traditional ethical guidance and expert opinions. A deeper engagement appeared to support greater operationalising of principles of inclusion, empowerment, and participant autonomy and supported researchers ‘ethical mindfulness’ which in turn may support instrumental outcomes of high recruitment, retention, and adherence levels.

**Supplementary Information:**

The online version contains supplementary material available at 10.1186/s12910-021-00740-1.

## Background

Research ethics sets out standards of research conduct based on a system of ethical values and guiding principles [1]. The application of ethical principles during the design, conduct and reporting of the research is intended to support the identification and management of a wide range of ethical issues, including protecting the dignity, rights, and welfare of any research participants [2]. Some areas of research such as biomedical research, present a complex and broad range of issues as it unearths ethical challenges [3]. The establishment of formalised ethical principles and general standards provides a structure for research practice [4], however, these guidelines are often unable to fully consider new ethical terrains that emerge during research practice. In these instances, researchers face challenges and questions that are unaddressed by existing standards [4]. Research dialogue and active participant engagement have been reported to promote discussions on values, norms, and virtues [5], creating a deliberative model for researcher and participant partnerships to resolve ethical challenges as they emerge [6], referred to as ‘embedded empirical ethics’.

## Approaches to embedded and responsive ethics

Several approaches to bring ethical processes closer to practice or to ‘embed ethics’ have been proposed. For instance, employing clinical bioethicists within hospitals, using ethics advisory bodies to support large research programmes, and conducting empirical ethics research alongside clinical and scientific research objectives. Conducting embedded empirical ethics research entails active involvement and dialogue with research participants, which can also be published as bioethics research contributions. It is regarded as a potentially powerful approach that (1) establishes a dialogue whereby participants and researchers engage through a deliberation, which helps to identify and raise awareness about ethical issues involved in research [6]; (2) facilitates ethical analysis and promotes discussions about appropriate courses of action [7]; and (3) employs social science methods to generate and analyse data about participants and researchers’ experiences and perspectives, collected before and during the research. project to inform an anticipatory and practical guide to good research practices [8]. The work presented here builds on many of these approaches to embed ethics by developing a method to facilitate ethical reflection and analysis within large research programmes. This is achieved by combining applied ethics and elements from technology assessment that can support real-time ethical mindfulness and co-production of inclusive processes [9, 10]. This approach is defined as a Real-Time Research Ethics Approach (RTREA).

Building on previous bioethics work, the RTREA is situated in a participant-researcher relational approach where (1) reflection on ethical principles and guidelines are continuously considered in line with the study and context, and (2) the realities of settings and participants’ experiences promote analytical decision-making. It differs from traditional empirical ethics approaches due to its features of continuous reflexivity, responsiveness, and disclosure [6]. The potential value of the RTREA lies in its ability to continuously reinforce the need to respect and protect study participants, to support the assessment of research stakeholders’ responsibilities and obligations [4, 9], and offer mechanisms that make appropriate use of evidence and traditional ethical guidance and expert advisory opinions to inform deliberation and decision-making [3].

In this paper, the researchers report how embedded ethics expertise can promote ethical practice and support ethical mindfulness within ANH research programmes. The extent to which the approach resulted in instrumental outcomes, such as participants’ enrolment, retention, and adherence, is also discussed. These reflections are drawn from a community-based, randomised, controlled trial, conducted in a rural area of Malawi titled ‘Addressing Hidden Hunger with Agronomy’ (AHHA) trial. The AHHA trial [11] is part of a project examining the potential benefits of agronomic micronutrient fortification. It involved distributing maize flour to study participants, with some receiving flour enriched with selenium and others receiving control flour [11].

## The AHHA trial

In 2019, the AHHA trial was conducted in Kasungu district in the central region of Malawi as part of a project involving the Lilongwe University of Natural Resources (LUANAR), the University of Nottingham, the London School of Hygiene and Tropical Medicine, and the University of Malawi, College of Medicine. These institutions partnered to conduct a community-based randomised trial to address micronutrient deficiencies, also known as ‘hidden hunger, that is widespread in Malawi [12]. The trial sought to test the efficacy of improving selenium status through the consumption of agro-fortified maize flour. Agro-fortification involves enriching maize—the staple food of Malawi—with selenium through fertilisers [13, 14]. The trial randomised 180 households each contributing one woman of reproductive age (WRA, 20–45 years of age) and one school-aged child (SAC, 5–10 years of age) to receive maize flour enriched with selenium (n = 90 households) or not unenriched flour (control; n = 90 households) [11].

One-hundred and eighty households participated in the trial, with households receiving enough flour to meet all their constituent member needs (i.e. 330 g/capita/day) for eight weeks. Maize flour (not enriched with selenium) was also provided to all other households in the study area to reduce the likelihood of participant households selling or donating their allocated flour to others [11]. The study activities included anthropometry, blood sampling and dietary assessment at baseline, distribution of the study flour during the intervention, adherence monitoring, and anthropometry, blood sampling, and dietary assessment at end-line.

## The elements of the real-time research ethics approach

The RTREA within the AHHA trial had several key features, namely (1) defining the role of the embedded ethics team, (2) facilitation of social interactions between key actors, (3) mapping of different knowledge sets and pluralistic experiences; (4) the application of ethical analysis to any identified issues, (5) ability to respond to ethical issues identified during the research activities in real-time (6) building and maintaining partnerships and supporting mutual trust between stakeholders. The BT emphasised a number of these features that were deemed to be critical when applying the RTREA from trial development to implementation in this study.

An important feature of the approach is the role of an (1) *embedded ethics specialist,* usually, a bioethicist or a bioethics team (BT) in ANH projects, who is responsible for facilitating dialogue, mapping ethical concerns and issues, conducting structured ethical analysis, and facilitating dialogue and communicating perspectives, issues, and concerns. The ethics team taking on this role must have independence from the wider research team. In this study, the BT included four experienced bioethicists from the College of Medicine and the University of Nottingham. The BT played an important role in interacting with all of the trial stakeholders (i.e. trial participants, participating communities, and the Trial Implementing Team (TImT)) and taking on the embedded ethicist roles. They acted as a bridge between TPs and the TImT.

The BT emphasised the following features that were critical in ensuring the success of the RTREA from trial development to implementation. (2) *Facilitating social interactions* among the potential TPs and TImT is a key activity. This study entailed gathering evidence to facilitate an understanding of ethical issues inherent in ANH research by gauging trial experiences and perspectives. The AHHA trial underwent formative research [15] one year before the baseline survey to gauge community perceptions of the upcoming trial activities and potential areas of concern to support prospective participants and increase the chances of a successful trial [16, 17]. The formative research provided valuable information on the social and contextual factors that can influence the uptake of study obligations and the researchers’ responsibilities in real-time.

The third feature (3) *focused on the mapping of different knowledge sets and pluralistic experiences* was operationalised by mapping TPs’ diverse knowledge and pluralistic experiences; and recognising social, ethical, and contextual aspects of the TPs experiences and their interdependence in real-time. These characteristics show a strong link with classical principles of “respect for persons” [1]. However, this notion went a step further to outline learning experiences, understand the dynamic process of decision-making, and focus on TPs’ understanding of the trial objectives and activities.

Applying (4) *ethical analysis to inform decision-making* is a key feature that supported decision-making. The BT employed an iterative process connecting the ethical analysis to the empirical research and the dialogue process. The team ensured that the analysis of empirical data and further ethical analysis and decisions were widely discussed and agreed upon by the stakeholders. This supported ethical mindfulness throughout the study.

In this context, the BT conducted empirical research simultaneously with the AHHA trial implementation to identify meaningful TP challenges and be (5) *responsive in real-time* as this trial was planned and conducted. This was achieved by soliciting feedback, achieving consensus, and aiding the design and implementation of the research. The aim was to take the priorities, interests, perspectives, values, norms, preferences, and welfare of TPs and participating communities into consideration.

The last feature focused on (6) *building and maintaining partnerships and supporting mutual trust between stakeholders,* which facilitated interaction between key research stakeholders in a deliberative process. The BT employed information-sharing sessions, and focus group discussions (FGDs) to facilitate dialogue and interactions. These efforts not only enhanced the informed consent process by providing alternative for information sharing but also fostered and encouraged skills and confidence to achieve self-efficacy and self-determination. This process recognised informed consent as a continuous process [18] and the BT facilitated the development of a responsive programme and study activities.

## Methods

### Study design

An action research project was undertaken from May to October 2019 to document and critically examine the implementation of the RTREA and map how the BT engaged with the Trial Implementation Team and TPs. This process was designed to improve research practice by exploring TP's perspectives and experiences, promoting evaluations and reflections on decision-making based on documented evidence and proposing new actions or adaptions in practice [19]. To provide evidence on broader contextual factors and to operationalise ethics principles, an empirical study was conducted to explore the experiences of TPs and the participating communities. The BT was embedded in the TImT to provide relevant expert advice based on the application of ethical principles and empirical experience.

The BT provided research ethics training to the TImT before the trial commenced. In support of the structured ethical analysis, the ethical principles of doing good, not inflicting harm, justice, respect for autonomy, alongside empowerment, social responsibility, participation, openness, and accountability were applied. A principle-based approach was used within this work as the most applicable ethical framework for informing the analysis of the ethical issues raised and it was also deemed to be in line with the approach used within REC evaluations that inform the ethical standards set for this type of trial. A principle-based approach is also aligned with research ethics standards that are most often used, as a guiding ethical approach, by researchers [1].

To support ethical mindfulness, decisions were made on social and ethical challenges that emerged throughout the design and conduct of the AHHA trial after a review of the ethical principles. A thorough assessment of the negative and positive impacts on TPs adherence, retention, and wellbeing was undertaken. The study aimed to answer the following questions: What would TPs’ lived experiences of the study be? (do no harm, do good)? How do we promote informed consent (respect for autonomy, openness)? How do we promote dialogue, voluntariness, and partnership (participation, empowerment)? How do we define and enact the responsibilities and obligations of the researchers and study participants (social responsibility, justice, and accountability)? We followed the RATS guidelines in presenting the manuscript including the results of the study (see Additional file 5).

### Study setting

The study and AHHA trial were conducted in Wimbe Traditional Authority, Kasungu District, in the Central Region of Malawi. The area is primarily characterised by subsistence farming, alongside smallholder and estate tobacco production.

### Sampling and selection of study participants

There were 12 villages in the AHHA trial study area. The TImT observed that negative rumours about the AHHA trial were circulating in seven of these villages before initiation of the trial, so the study recruited from these villages. The sampling process was initiated by convening a meeting with the AHHA trial manager to collect and analyse data following the research team’s interactions and engagement with the participating villages during the AHHA trial baseline survey. Data collected during informed consent observational meetings were analysed to help map social and ethical issues that required further exploration in subsequent data collection activities.

### Selection and recruitment of study participants and their partners for FGDs

The BT purposively recruited TPs and their partners for FGDs. To improve the heterogeneity of the sample, TPs who had school-going children were recruited. This permitted the BT to explore a wide range of TPs’ experiences. In total, 71 TPs took part in the first round of FGDs. To maximise recruitment effectiveness, each participating village had volunteers who approached all potential TPs from their respective villages [19] to participate in the interviews. On the appointed day, the BT member and the research assistant approached the TP and their partners in schools, church grounds, community clinics, and flour distribution centers. TPs and their partners were informed of the study, and those willing to participate were called for informed consent procedures and discussions. The inclusion criteria for study participants were: TPs and partners willing to participate in FGDs; the family had a school-going child who donated blood; and was willing to participate in the second phase of data collection. TPs with school-going children were recruited so that they could share their children’s experiences. TP partners described the various experiences they encountered within the community due to their partner’s involvement in the trial.

### Pre-FGD session, self-assessment for TP and their partners

The BT member and research assistant held a 40–60-min informative discussion session with TPs and their partners before data collection commenced. This session was conducted during the first phase of data collection, and after each session, all TPs and their partners were called to attend the FGD. These discussion sessions were offered in a space that allowed free conversation on various issues.

The session was designed to share knowledge and assess study participants’ ability to offset myths and misconceptions about the study, report serious adverse events, ask and share information with others, and assess their information needs. Discussion topics included informed consent, assessing focused interactions between the TImT and the TPs, TPs’ knowledge about the trial and general information about researchers’ obligations, research governance in Malawi, and the role of the BT (see Additional file 6). It was emphasised that the BT was a bridge between the TP and the TImT, and TPs were encouraged to share their wider trial experiences with the BT.

At the end of the session, an assessment quiz was conducted to gauge TPs’ understanding of the trial and record areas that needed further clarification. The results were compiled with other reports and widely shared with the TImT for their action.

### Selection and recruitment of key informants (KI) for in-depth interviews (IDIs)

Key informants were selected based on their roles and responsibility in the community to gather their perspectives of TPs’ various trial experiences. The BT and research assistant recruited seven key informants: one local chief, two volunteers, one religious leader, one village committee member, and two health surveillance assistants. The local chief was chosen as a gatekeeper who would be trusted by the TPs with information about their concerns, fears, and trial experiences. The BT assumed that the local chief oversees the welfare of various communities, representing a good source of relevant information about the trial. Similarly, two health surveillance assistants were chosen—one female and one male—to represent the TPs and their partners, and provide relevant safety information from their point of view. The religious leader was chosen as they would provide a religious point of view. The eligibility criterion for key informants required their availability in the community for the duration of the trial. All IDIs with key informants were conducted at their homes.

### Data collection for FGDs

A female research assistant with knowledge of research ethics was recruited to assist the BT with data collection. Her knowledge of research ethics supported the mapping of ethical and social issues. The research assistant received training to familiarise herself with the process of data collection and the RTREA.

To facilitate their understanding of the study and its procedures, all participants received an information sheet to read carefully. The information was read to all TPs who could not read. Participants were reminded their participation was voluntary and would not impact their involvement in the AHHA trial. They were also reminded that their information would be digitally recorded for accuracy and completeness, which all TPs accepted. To enhance TPs’ privacy, all participants were assigned a unique number for identification. The FGDs were moderated by a BT member and the research assistant. All non-verbal behaviours during FGDs were also recorded.

Apart from the longitudinal data collection, FGDs were collected over two phases: the first phase was after the baseline survey, before initiation of the intervention, and the second phase was before the end-line survey, six weeks after the initiation of the intervention. The BT intended to conduct six to eight FGDs in each phase and recruit a maximum of 8–10 participants per FGD. One of the challenges of collecting longitudinal data is the need to ensure participant retention [20] and thus recruit sufficient participants per FGD (8–10 participants) and strictly check the inclusion criteria with TP.

**Fig. 1:**
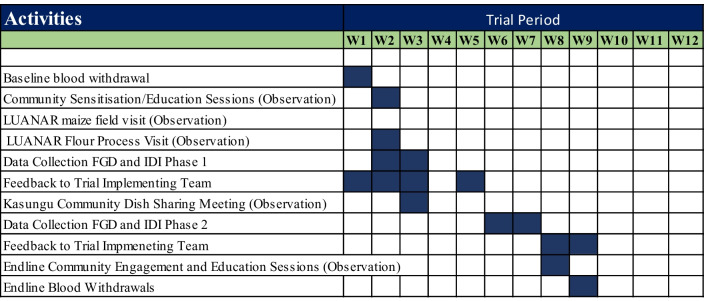
Data collection time points, reporting, and study activities (weeks 1–12)

All FGDs were conducted in the local language, Chichewa. A pre-tested unstructured discussion guide was used and had a broad question, namely: “what are your trial experiences?” Probes were used during the discussion to obtain detailed and relevant information about their overall experiences and information needs (see Additional files 1 and 2). For example, participants were asked to suggest how their trial experiences could be enhanced, and what the TImT should focus on. The unstructured discussion guide revealed greater insights into TPs’ experiences within their communities, since prior information from reports could not reveal real-life experiences. Therefore, the participants’ narrations shaped the interviews. After the unstructured discussion guide was used, an open-ended interview guide was developed that focused on the following topics; perceptions about the AHHA trial, (2) myths and misconceptions about the trial, (3) perceptions about the informed consent process, (4) information needs of the TPs and wider community, (5) perceptions on the roles and responsibilities of TPs and the wider community, (6) decision-making norms, and vii) questions to understand everyday experiences in the trial. The interview guide was in Chichewa and translated to English. After each FGD, a report was shared with the research assistant to compile a single report that was widely shared with the TImT for their action. Additionally, the report was reviewed for categories that would inform the development of the next set of questions for the second phase of data collection. Refer to Fig. 1 below for data collection time points.

Demographic details collected from phase one included age, qualification, marital status, prior recruitment in clinical research). The age of participants was between 35 and 50 years, and the majority had a primary school education. Most participants were farmers. During phase one, each FGD lasted two hours.

For phase two, FGDs were held with the same set of TPs who were interviewed during the first phase. The second phase interviews explored TP perspectives of the AHHA trial, identified facilitators to treatment adherence, TPs perceptions on flour safety, dealt with negative trial experiences like rumours, and inquired on social and ethical issues as defined by the TPs.

### Data collection for KI IDIs

The BT member and the research assistant conducted all key informant IDIs using open-ended questions (see Additional files 3 and 4). The IDIs were conducted in Chichewa, and each interview lasted 40–60 min. All participants consented to having the interviews recorded. Fourteen IDIs were conducted with local chiefs, volunteers, TPs, and religious leaders who were purposively selected [18], focusing on maximising diversity, social interaction status, and responsibility within the community. Participants’ responsibilities and roles concerning the AHHA trial were also a consideration. As with the FGDs, the IDIs focus was on the following issues: (1) perceptions about the AHHA trial, (2) myths and misconceptions about the trial, (3) perceptions about the informed consent process, (4) information needs of the TPs and wider community, (5) perceptions on roles and responsibilities of TPs and the wider community, (6) decision-making norms, and (7) questions to understand everyday experiences in the trial.

### Structured observations

Observations are an important part of data collection, especially in action research [20] where data collection within research environments is required in real-time as they are experienced. An observation schedule was developed that allowed the researchers to record actions and interactions between TPs and the TImT. The researchers captured relevant behaviour, actions, and events in a systematic manner [19]. This data collection stage involved assessing TImT and TPs discussions about their fears, concerns, asking and answering questions through various study activities that were organised by the TImT. The findings resulted in more focused and detailed information to clarify the study procedures and build trust [21].

The researchers conducted five structured observations during the AHHA trial (Table 1). On average, observations lasted between one to two hours.

**Table 1:** Number of interviews/activities conducted at each time point

Total number of activities/interviews	During enumeration	Community sensitisation meetings	Baseline study (before intervention, after 1st blood donation)	Before flour distribution	After intervention (flour consumption period)	Endline study (before 2nd blood donation)	Total number
Observations	0	1	1	1	1	1	5
Reports	2	5	2	1	3	2	15
In-depth interviews	0	0	7	0	0	7	14
Focus group Discussions	0	0	5	0	0	5	10

#### Trial reports

The development of FGD and IDI guides was mainly informed by the analysis of data from study reports and structured observation reports. The researchers analysed 15 trial reports produced by the TImT, drafted after activities conducted from the inception to completion of the AHHA trial. For example, identification of rumours and the potential impact they may have on the trial emerged from the previous formative study and other trial reports, so this resulted in the development of interview questions related to these challenges.

#### Data management and analysis

All interviews were audio-recorded and transcribed verbatim by two transcribers based in Malawi, hired by the research team. The transcripts were read and checked during the data collection exercise to obtain an overview of the information and reflect on the meaning and impressions of their overall experiences and perceptions of the AHHA [19]. Actions, experiences, and events were recorded through memos [22]. At each stage, the researchers coded the data to start mapping the overall experiences, perceptions, and outline events that unfolded. The researchers also started to examine the interactions and actions that emerged following various activities that were implemented in the overall study. The initial findings from the interviews were fed back to the TImT through reports. Following the first round of analysis, a coding dictionary was developed that focused on the decision-making triangle (DMT) concepts. The data set was managed using NVivo 12.0 software.

#### The decision-making triangle framework (DMT)

Data analysis was guided by the DMT framework [23] which has been proven as a useful tool that combines evidence and ethics for decision-making. The pinnacle of the framework lies in the reflection of ethical principles, thereby promoting structured ethics analysis. The BT collected and analysed data while applying ethical principles to the findings. The triangulation of various data sources—as evidence of various social and ethical issues that emerged in the trial—and the utilisation of ethical principles informed the BT’s decision-making.

The DMT includes three constructs that promote ethical decision-making and consideration of implications for practice and how actors might respond. In terms of the RTREA’s application, the following was considered; (1) Researchers should decide how to achieve desired health outcomes and reduce harm. (2) In making such decisions, researchers should apply a set of principles that reflect their purpose, values, and obligations. The principles of respect for persons, confidentiality and privacy, informed consent, and non-maleficence (1) were widely applied in the AHHA trial when responding to social and ethical issues that emerged. (3) In applying these principles, researchers should make appropriate use of available information and examine the nature of actions and plausible responses. The framework promotes an iterative process that facilitates interaction, dialogue, and the inclusion of all research stakeholders. The BT iteratively approached data analysis; coding presented an opportunity to deductively [24] assess ethical principles and concepts from empirical ethics using the DMT constructs. As additional codes emerged, the researchers were able to inductively determine the principles at stake and gauge their analytical decision-making.

The BT extensively recorded their reflections, observations, research processes, and procedures throughout the study. Data were analysed using thematic content analysis [25] with the assistance of the DMT framework to provide information related to the RTREA applicability in ANH research.

#### Ethical considerations

In this study, the ethical responsibilities were above minimal risk as the study enrolled women in a community setting. The study obtained ethical approval from the College of Medicine Research Ethics Committee (reference number P.03/19/2633). Ethical approval for the AHHA trial was obtained from the College of Medicine Research Ethics Committee (reference number P.11/18/2539) and the LSHTM Interventions Research Ethics Committee (reference: 16181). The researchers used the TImTs sensitisation meeting platform to recruit potential study participants. The trial sensitisation coordinator introduced the study before any data collection commenced and all participants in the ethics study provided written informed consent. This approach enabled the researchers to clarify the main objectives of the ethics engagement initiative. To enhance comprehension, information sheets were shared and those who could not read were asked to bring a witness. Voluntary participation was an important ethical consideration and participants were reminded that their participation would not affect their involvement in the AHHA trial. The anonymity and confidentiality of TPs were also considered. FGDs and IDIs were conducted at school blocks or village court grounds, sufficiently removed from village households to avoid privacy concerns.

Asking participants to discuss their existing perceptions about the trial and its activities could cause them stress and fear. The TImT, therefore, explained the role of the BT, and before any FGD or IDI session, participants were informed that these were safe spaces in which they could openly share their experiences and perceptions. Participants were told that this would help the team identify and respond to potential issues [20].

## Results

Before discussing the implications of applying a real-time ethics approach for ANH research programmes it is important to set out the range of findings from the embedded activities. Operationalising the RTREA.

### Operationalising the RTREA

The RTREA was operationalised and embedded within the AHHA trial, in several ways. The RTREA was used to create spaces and opportunities to capture a rich array of data relating to the ethical implementation of the trial and facilitated participatory assessment. Using this approach the TImT reviewed reports and the analysis from the BT before conducting normative evaluations on their course of action which was drawn from the various recommendations/suggestions from the TP. The RTREA created an evidence-based (refer to Table 2) that the TImT used for ethical analysis. Various deliberative methods were employed between the trial implementing team, the BT, and TPs to gauge TPs’ preferences, values, and weigh principles that conflicted with each other, and understand their context.

**Table 2:** An outline of activities facilitated by the Bioethics Team and the recorded outcomes

Date/time	Activity	Task	WHO	Issues	Data type	BT responsibility	Outcome
Annual Planning workshop October 2018	Face to face interactions by trial implementing team	Facilitate discussion to identify ethical issues with the overall study activities	Bioethics Team, Laboratory Team, Soil Experts, Trial Management Team, Economists	Patient safety, Informed Consent procedures, study design, compensation, community engagement, maize distribution	Ethno-Notes	Facilitated discussion of impact of study design and activities, Discussion on safety and informed consent procedures	Development of safety reporting tools, development of an educational community engagement process, training on ethics to the study team, development of Informed consent evaluation strategies
Formative Research	Survey or questionaires between potential trial participants and trial team members	Gauge community perceptions and potential areas of concern	6 FGD, 7 In-Depth Interviewa with community members	Family planning concerns leading to infertility, fears regarding blood sampling and witchcraft and social stigma	Interviews	Ensure comprehension of study aims and procedures	Edit Protocol, Incorporate real time ethics approaches
Maize Field Visit April 2019	Face to face interactions with community representatives	Gauge community perceptions and potential areas of concern about the maize production procedures	Community respresentatives, LUANAR team members and the ethics team	Rumours about maize field, growing protocol, fertility issues, double blinding procedures, flour consumption	Ethno-Notes	Feedbac to Trial implementing Team on TPs experiences, concerns and perspectives	Develop community education sessions
Baseline Study July 2019							
Community engagement and Information Sharing session July 2020	The face-to-face interactions with wider community members	Enforce honest discussion, facilitate interactice process between communities and trial implementation team	Community members, LUANAR team members and the ethics team	Safety of flour and flour consumption procedures. To higlight milestones on study activties and reminder on study roles and responsibilities	Ethno-Notes	Promoted consistency, Cemented trusting relationship	Developed community engagement approaches and enhanced informed consent frameworks to highlight participants roles and responsibilities, address concerns and myths. Exist strategies
Flour Processing Visit July 2019							
Dish Sharing get together meeting August 2019	The face-to-face interactions with community representatives	Gauge community perceptions and potential areas of concern about the flour production process	Community representatives, LUANAR team members and the ethics team	Mechanism to nullify rumours, alleviate fear about the flour and cement trust	Ethno-Notes	Facilitated undertanding of information needs, safety concerns	Alleviation of fear, addressed safety concerns, cemented trusting relationships, enhanced informed consent, increased study roles and capability
Endline community Information sharing session Sept 2019							
Endline Study Sep 2019	Active dialogue between Trial Participants and the Ethics Team	Assess ethical and societal issues of the AHHA trial and determine best practises for respect for persons	5 FGD, 7 In-depth Interviews with community members	Blood donation procedures, trial access, compensation rewards	Interviews	promoted ethical mindfulness on how to share study results	Development of study results dissemination protocol

### Mapping and analysing key ethical challenges

Based on the features of the RTREA, the DMT constructs (Table 3) were applied to identify and analyse the key issues. Codes were then organised into categories and themes, reported as results. The analysis reflects five major themes; (1) Responsive and constructive approaches to tackling diverse ethical issues during research implementation. (2) A catalyst for participant engagement and reflections on participants roles (3) A catalyst for enhanced informed consent (4) A catalysts for participant empowerment; and (5), Acknowledgement of study participants’ world. The themes are discussed next.

**Table 3:** Development of themes using the DMT concepts

DMT constructs	RTRE approach (AHHA trial decisions/outcomes)	Key themes
Ethical principles: Do good, do no harm, empowerment, social responsibility, participation, openness, accountability, fairness	Education sessions with Trial Implementation Team and TPs	Responsive and constructive approaches to tackling diverse ethical issues during research implementation A catalysts for participant engagement and reflections on participant roles A catalyst for enhanced informed consent
	Maize field visit, flour processing visit, dish sharing get-together meeting, Tailored information education sessions	
	Recruitment of community volunteers	
	Engagement with local leaders	
	Ethics workshops, ethics approval, study protocol	
	Informed consent, information sheets	
Evidence	Focus groups discussions	Acknowledgement of study participants’ lived world
	Trial reports	
	Observations	
	In-depth Interviews	
Suppositions on appropriate actions	Delivering Respect for autonomy	A catalyst Enhanced Informed consent
	Supporting Voluntariness	
	Respect for persons	
	Protecting Privacy and confidentiality	
	Enhancing Safety	
Decisions	Study design and trial activities, the safety of study participants, community engagement, strategies to enhance positive experiences, trial results dissemination protocol, community exit strategies, ethics training	A catalyst for participant empowerment

To promote ethical conduct during the AHHA trial, decisions were made to enhance informed consent, offset social harm, and improve TPs’ experiences. These included ethics training for the Trial Implementation Team, formative research processes, ethics workshops, and various trial activities that were implemented widely, refer to Table 2.

### Responsive and constructive approaches to tackling diverse ethical issues during research implementation

The RTREA supports the embedding and real-time awareness of ethical principles. For this trial the need to protect and respect study participants’ welfare, support researchers’ wellbeing by providing a mechanism to discuss and analyse potential challenges, and promotes scientific integrity. These were represented in the AHHA trial by the decision to incorporate the RTREA. RTREA offers a robust, real-time engagement that includes investigation of ethical issues through the collection of data that served as evidence of emerging issues to help inform deliberations and decision-making.

Several social and ethical concerns were identified through FGDs and IDIs. Study-related anxieties and rumours that emerged included, (1) blood drawn from participants will be sold, (2) participants who donated blood will die, and (3) men will have fertility issues after eating fortified flour. Challenges were also identified relating to social interactions, community randomisation misconceptions, therapeutic misconceptions, perceptions about compensation, voluntariness misconceptions, and perceptions about the return of study results. These barriers and facilitators presented certain implications and research obligations.

Further evidence that informed the mapping and analysis (part of the DMT model) of ethical issues was collected as issues emerged. TPs were asked to reflect on their overall study obligations while the TImT were simultaneously being asked to be mindful of their role and how their responses may impact wider research findings. In line with the DMT model, evidence was collected to further assess TPs’ positive and negative trial experiences regarding the wellbeing of participating communities. For example, it was made clear that researchers have an obligation to disseminate research results:

…The main thing that we expect is that when this program is completed we should see the outcome (FGD 201, Phase 1, Study participants and their partners).

However, it was unclear how researchers ought to carry out this obligation. In some research contexts, especially in community settings where studies are engulfed with negative experiences (such as rumours, myths, and misconceptions), the way results are shared can render TPs vulnerable to social harm. In this setting, TPs expressed concerns that the results of the trial might have a ‘null effect’, and this may be seen as a form of failure on the part of the participants as expressed below.

… It would be a very proud thing if what the Bunda people want in our bodies worked. But if it would not work then it would be worrying because then the ridicule we are receiving from people will never end. But if it is accepted that they have found the results they wanted then we would have answers for those who ridiculed us. (FGD 304, Phase 2, trial participants and their partners)

.. The other question is the same one, that will they just leave us that the research has ended today. The researchers will say bye! Bye everyone and not come back? There the fear is that when we stop eating this flour and have met problems how will they know that we have met these problems? Will they continue visiting us when we change your food that we were eating and start eating our own food or is it the friendship of the axe? (FGD 305, Phase 2 Study participants and their partners)

### A catalyst for participant engagement and reflection on participants’ roles

The RTREA acted as a process to support the role of the BT as a knowledge broker [26], investigating information needs and promoting interaction between TPs, participating communities, and the TImT, as well as facilitating the development of strategies to expedite the uptake of study roles and responsibilities. The BT used their knowledge from empirical research on ethical challenges in conducting studies in low-resource settings [27], ethical issues emanating from misconceptions of enrolling in research without due consideration of risks associated with study participation [28], and informed consent procedures—specifically the need to understand how well negative feelings are addressed, and how well cultural aspects are managed [29]—to understand how these could be addressed during the AHHA trial’s implementation. Additionally, the researchers explored how personal interactions are believed to influence research participants’ understanding and the values of TPs’ experiences [30, 31].

An assessment of the broader contextual factors is crucial, where there is limited knowledge of clinical research, the enrolment of minors, vulnerable communities, research that can fuel myths and misconceptions, and various beliefs about blood. Constructive feedback on the trial process was largely encouraged by the BT as this information was crucial in identifying how important ethical principles may be infringed on. In turn, these factors could promote or hinder trial participation, adherence, and study retention. Being embedded enabled the BT to facilitate discussions without compromising stakeholders’ relationships. ‘Conversation spaces’ that were created by the BT highlight the importance of the interpersonal, interactive, and social skills required to open ‘black boxes’ of trial issues or facilitate discussions on sensitive topics. The RTREA helped the TImT develop strategies that promoted their mutual understanding of challenges and helped them develop approaches to address these difficulties.

### A catalyst for enhanced informed consent

Various ethical principles were evident in the trial. The RTREA provided mechanisms that offered opportunities to engage and gauge study participants’ information needs. Various information assessment methods were employed to identify information needs at particular points in time, thereby establishing a systematic way to promote openness, accountability, and encourage participation. The BT was solely involved in appraising and identifying implications of information in line with the needs or demands of local contexts. The RTREA provided a conducive environment where TPs were open about their safety concerns, fears, and misconceptions, and the TImT responded to negative experiences by developing tailored information-sharing sessions.

The RTREA supported the process of enhanced informed consent and recognised the importance of creating an open encounter with the BT independent of the TImT. To increase participant engagement, the environment provided opportunities to share trial experiences, ask questions, suggest solutions, seek information, discuss respect for community systems, and engage key community gatekeepers:

… The issue is that we would very much like that our colleagues who are explaining these things should not stop coming. They should be coming now and then, teaching those people that are left behind to join the other group because some were left behind indeed, they did not understand. So we were thinking that these meetings should continue happening now and then up to the time we are about to receive so that those people should be taught and should know where they are coming from and where they are going (IDI 102 Male Volunteer, Phase 1)

### A catalyst for participant empowerment

The principle of empowerment was represented through the procedures that were implemented in the RTREA with the main aim of empowering TPs. were designed to promote mechanisms that seek to capture and consider TPs’ perspectives and experiences through various participatory methods that were widely implemented during the AHHA trial; namely, workshops, IDI, FGD, field visits, sensitisation meetings, and dining together. This enabled a trusting relationship to develop through spontaneous conversations and exchanges of information. The task of tailoring information according to needs, making it understandable and useful, required a facilitator to capture and gauge the relevant information being sought; the BT took this role. These experiences were fed into the TImT process by the BT through reports developed from the IDIs and FGDs. The approach highlights the significance of responding to information needs by being mindful of the context, emerging perspectives and misconceptions that may hinder or facilitate participation and compliance. Although study participants were equipped with information sheets, the participatory approaches were well deemed to be important spaces as expressed by the TPs.

The RTREA involved the trial participant-centered process where TPs and participating communities were placed at the centre of the dialogue and knowledge exchange process, with various learning activities [e.g. (1) flour processing tours, (2) maize growing field visits, (3) dining together], so they were felt equipped to engaged and were more motivated to seek information to understand, refute or address negative experiences. This approach highlighted how study participants were able to express their concerns as well as challenge their own and other people’s misconceptions and reason through them:

… For us to be strong-willed to see this research through to the very end it is because we are brave right from the start. Whatever people may say but we still want to see how it ends because we know its importance since these people visit us very often … They want to find salts in our bodies so that they add to fertilizer. Those people who visit us ask us what changes we see in our bodies or the problems we have with it or how we have embraced it. So we say that we do not have any problem with the flour. They tell us they took our blood so they can differentiate the zinc salts in our bodies before we started eating the flour and after we started eating the flour. That is why we became strong-willed so that we should see the end. We are not going back, we volunteered. (FGD 304, Phase 2, Study participants and their partners)

The BT’s immersion in participating communities illuminated relevant information needs and an understanding of their values and preferences. The enhanced engagement did not only reveal relevant information needs but provided the ability for the researchers to be more mindful of the need to develop trusting relationships and share relevant information. In this study, the TImT was challenged to question their ethical competence by reflecting on the principle of privacy and confidentiality that was at stake and weighing these against the study context, the preferences of the TPs, the need to create trusting relationships, and information sharing. It was also crucial that the TImT considered the consequences of not sharing the relevant and useful information as required. This was a case of keeping any adverse events confidential and private versus publicly sharing the clinical outcome of the event to help alleviate the fear of side effects, refute rumours, and build trust. Ultimately, TPs preferred openness.

### Acknowledgement of study participants’ lived world

An important aspect of being responsive and reflective is to identify evidence (one of the major pillars of the DMT model) that can be taken into account. There is a need to detail what impact the intervention may have and who will have to bear any consequences, hence the need to present and use appropriate evidence. One of the key features of the RTREA depends on the ability to acknowledge and reflect on participants’ trial experiences and consider the impact on their lived experiences which, in return, can affect compliance, retention, and safety. This approach helps trial researchers develop strategies to offset and maximise positive trial experiences by responding to collective and individual values, norms, beliefs, and needs.

By continually assessing TPs’ experiences, it was noted that the language and words used within the communities had an impact on facilitating or hindering TPs’ responsiveness to trial obligations (See Table 4 for details).

**Table 4:** An outline of words reflecting on the implied impact of the uptake of study activities

Word	Data Collection Phase and Method	Deliberated Impact on study activities
"Chitonzo" "ridiculed"	FGD Chimsekesa Phase 2	Concern/consequences of sharing negative results to the wider community
"Kupopa" "'sucking"	FGD 203, Phase 1, Trial Participants and partners	Exaggerating blood volumes being donated. Concern on blood donations
"Chikondi cha Nkhwagwa chokoma pokwera'" "The love of an axe sweet during climbing"	IDI 101, Phase 2, Trial Participants	Post Trial access to food and concern about exploitation
"Kuchirandira" "Receptive"	FGD 203, Phase 1, Trial Participants and partners	Misconceptions about voluntariness
"Ufa ndi moyo" "food is life"	FGD 305, Phase 2, Trial Participants and partners	Theurapric Misconception
"Kuzipereka" "Special"	FGD 304, Phase 1, Trial Participants and partners	Misconceptions about voluntariness
"Mwabetsa" "Sold"	FGD 203, Phase 1, Trial Participants and partners	Mockery concerning blood donations

A thorough reflection on these words and encounters helped the TImT to be mindful of their communication strategies. For example, visual aids (vacutainers for blood donations were shown to communities during sensitisation meetings) were employed to provide a visual presentation of tools that were verbally discussed. Also, tailored information supporting the formal consent and participant information sheets was widely shared to clarify and address various misconceptions around blood.

## Discussion

Operationalising a concept of the RTREA is a new approach, and it can also be argued that its application in ANH research to investigate ethical issues as they emerge in real-time throughout the research is novel. It appears to be a valuable and effective mechanism to reflect on ethical principles and guidelines in line with initial study planning alongside REC requirements, as well as the wider context of research ethics [6]. The RTREA appeared to enhance issues that are important in developing and conducting robust ethical research practices. These include mindfulness through the responsiveness to ethical and social issues which are analysed through the application of ethical principles and operationalised in line with ethics guidelines but specific to the study context. The findings indicated that ethical guidelines and norms specify researchers’ obligations; for example, respecting study participants by sharing results [30, 31]. However, this process also helps researchers apply potentially more meaningful and robust ethical practices that enhance trust and notions of the research partnership. Researchers should reflect on their ethical competence by acknowledging the ethical dimension that informs the obligation for good research practice. They should also have the capacity to think through and respond appropriately to challenges [9]. This approach provided an opportunity to understand the context, discuss social and ethical issues, and establish measures to protect study participants from potential social harm [32].

Formal reflection sessions to identify, define, and discuss trial experiences with TPs and key stakeholders created ‘conversation spaces’ [26] that opened the ‘black box of ethical dimensions inherent in ANH research. These spaces are intended to be utilised with existing structures within the communities and are solely driven by continuous dialogue and learning, which comes from stakeholders. Creating discussion sessions and interactions are crucial activities to support participation and fully enact ethical principles to deliver real-time responsiveness. As Lorraine [27] suggested, a participatory process helps to raise and resolve ethical dilemmas. Thus, engaging study participants in participatory research—where they take an active role in discussing study experiences, share various perceptions, and suggest solutions to ethical dilemmas—where their input is written up, helps researchers not to forsake their ethical obligations but rather provides an opportunity that allows them to be mindful in offering solutions [7].

As a novel approach, the study findings suggest that the RTREA highlighted the importance of interactive sessions encouraging and promoting discussions with different stakeholders while considering group dynamics and information needs [33]. The BT was seen to promote the ethical obligation of achieving informed consent [33] by providing additional support and relevant information, while simultaneously offering various insights on what relevant information was required by TPs and why. This helps researchers go beyond any possible assumption about how their protocols are working in practice and how they are supporting informed consent by presenting real-time evidence on TPs experiences. This approach supported TPs’ empowerment by allowing them opportunities to ask questions, regarding TPs as research partners rather than passive recipients of research procedures [34]. More specifically, in identifying information needs, this approach revealed prior knowledge or personal experiences which are critical when constructing new knowledge. Learning encounters for adult learners should employ interactive activities and processes, which were reported as being achieved by engaging TP/participating communities in education encounters where discussions and question-and-answer sessions took place. Also, the learning needs of adult learners should be investigated, which was realised by exploring the information needs and trial experiences by inquiring and compiling a list of rumours, myths, and misconceptions to help the TImT develop tailored messages to respond and refute them. Finally, by ensuring that existing frames of reference have been used, as demonstrated in other studies, an understanding of study concepts, designs and activities were enhanced; as realised by prior knowledge and observed life experience [30, 34, 35].

This study also demonstrates that the RTREA allowed the TImT to take empirical information related to TPs’ perspectives and knowledge seriously in an attempt to understand the cultural context, talk with participants, and make genuine efforts to understand the TPs experience as well as what can potentially facilitate or hinder enrolment, compliance, and retention. The BT facilitated key communication practices that include both inquiring and informing to assist participants in making decisions about trial involvement [35]. Informing involves providing the participants or participating communities with evidence and additional information about the study’s objectives, safety procedures, benefits of compliance, and consequences of non-compliance [35]. Inquiring was considered helpful in assessing the TPs’ knowledge, expectations, fears, and beliefs that may have been derived from lay networks or other information sources that could have impacted recruitment, retention, and compliance [33, 34]. The provision of information, therefore, empowers TPs to make informed decisions about trial participation.

In this study, the RTREA facilitated TPs’ involvement and encouraged them to share their lived experiences. Various engagement sessions increased opportunities to explore trial experiences, discuss ethical approaches in undertaking research obligations versus reflecting on consequences, or the impact of trial obligations [7]. The RTREA may be beneficial as it provides a process that maps and then shares some of the complex aspects of participants’ ‘lived experience’. Any understanding of a participant must be profiled in real-time so that the concept has meaning. Strategies to ensure that staff remain motivated and informed on how best to protect TPs included ‘iterative dialogue’ with feedback from the BT to support their abilities and needs to judge the ethical acceptability of various aspects of the trial. However, by keeping TPs’ lived experience at the centre of this approach, the RTREA did not only enhance informed consent but also allowed the TPs to take ownership of their study responsibilities and develop self-efficacy to refute, address misconceptions, and reason through them.

It is important to recognise the positionality of the BT as they worked to facilitate the RTREA and support various stakeholders, while still being part of the wider project. Although such positionality presents some risks, the interactions with the key informants, the structured use of formal report writing, the clear protocols for focus groups and interview schedules, the review of this work by the local REC and the prominent role of ethical principles in this work, provides clear standards for the BT and in turn, should underpin the integrity of their work. Although further work can be done on the issue of positionality, the participatory methods used within the RTREA were reported by Oviedo-Joekes et al., to have enhanced informed consent [14], foster the feeling of collective ownership [6], and provide a research setting that is respectful and uplifting of TPs’ rights and welfare.

Participants’ autonomy was assessed to determine how personal factors—such as relationships with significant others, the environment, personal beliefs, life experiences, prior knowledge, historical context (e.g. previous research activities, relationship with regulators/key gatekeepers) -impacted TPs’ uptake of trial roles and their sense of obligation and responsibilities. Thus, as presented above, the TImT’s communication qualities were critical in providing relevant and consistent information and offering a conducive environment for ‘dialogue.

## Conclusion

This paper has indicated the possibilities of RTREA for researchers and research activities, especially for research being implemented in low-resource settings. The RTREA provided a mechanism that raised awareness and facilitated the identification of ethical challenges inherent in ANH research, reflected the anticipated social impact of the research, and acted as a process to support education, interpretation, and facilitation. It can be argued that the RTREA supports both researchers’ and study participants’ wellbeing by providing a mechanism to discuss and analyse potential ethically challenging issues during the time course of the project, support and promote scientific integrity of research endeavours that can lead to more defensible research outcomes.

RTREA integrates processes that foster dialogue between participants and researchers, prompting the development of shared understanding to improve good research practices. It may be argued that operationalising this approach through participatory methods that create ‘conversation spaces’ results in an interactive process that promotes common learning and understanding between stakeholders.

The RTREA reflected the value gained from enacting continuous and direct engagement with TPs and researchers on social and ethical issues, a process that can be used alongside traditional ethical guidance and expert opinions. This work revealed that AHHA TPs value a deeper engagement with the research community beyond what is formally required and signed off by RECs for study recruitment and participation. As an instrumental outcome of this work, this deeper engagement appeared to help achieve greater recruitment, retention, and adherence levels, which were beneficial in terms of the AHHA trial’s research objectives. Further research is needed and planned to develop this approach and examine the opportunities and the threats to the use of embedded real-time research ethics tools for ANH and other research fields.

## Supplementary Information

**Additional file 1**. Focus Group Discussion Guide Final 2019 Participants Phase 1.

**Additional file 2**. Focus Group Discussion Guide Final 2019 Participants Phase 2.

**Additional file 3**. In-Depth Interviews Key Informants Phase 2.

**Additional file 4**. In-Depth Interviews Key Informants Phase 1.

**Additional file 5**. 3 RATS Checklist Limbanazo Matandika Nov 2020.

**Additional file 6**. Self-Efficacy Assessment Tools for Study Participants 2019.

## Data Availability

The data that support the findings of this study are available from CEBESA but restrictions apply to the availability of these data, which were used under license for the current study, and so are not publicly available. Data are however available from the authors upon reasonable request and with permission of CEBESA.

## Abbreviations

COMREC: College of Medicine Research Ethics Committee
FGD: Focus group discussion
IDI: In-depth interview
REC: Research Ethics Committee
BT: Bioethics team
TP: Trial participant
RTREA: Real-time research ethics approach
AHHA: Addressing hidden hunger with agronomy
ANH: Agriculture-nutrition-health
TImT: Trial implementing team
