# Supplementary material for: Operationalising a real-time research ethics approach: supporting ethical mindfulness in agriculture-nutrition-health research in Malawi
Source: BMC Med Ethics. 2022 Jan 11;23:3. doi: 10.1186/s12910-021-00740-1 (PMC8748184; doi:10.1186/s12910-021-00740-1)
Supplement: Supplementary file 1 — Additional file 1. Focus Group Discussion Guide Final 2019 Participants Phase 1. [file 12910_2021_740_MOESM1_ESM.docx]

1. **Knowledge and experience about research**
2. May you please tell me what you know about research? [ probe about design, study activties, benefits]

Mungandifotokozere kuti kodi kafukufuku ndi chani? [anali otani, Zochitika za mu kafukuku, phindu lakafukufuku]

1. Can you tell me if you have ever had a role in any research prjects?

Munayamba mwakhalako ndi udindo mu zochitika za mu kafukufuku wina aliyense?

If Yes: Prompt about previous his role involvement, significance, overall experinece or any concerns from previous research

Ngati ndi choncho: Funsitsitsani [zokhuza mitundu ya akafukufuku a m’mbuyomu, udindo wawo, Kufunikira kwa udindo wake, kapena nkhawa zina zochokera ku ma gulu a kafukufuku/ ma pulojekiti ammbuyomu]

1. **Understanding of present research**
2. How do would you describe how people in this community understand this research project ? [ probe: benefits, study activties, risks of taking part, why the village was selected]
3. Mungafotokoze bwanji za mmene anthu ammudzi akumvetsera za kafukufuku ameneyu?

Zotsatira za kafukufuku akatha

Mphindu lotenga nawo mbali

Phindu lakafukufuku

Zochitika mukafunukufuku

Kuopsa kutenga nawo mbali

Masankhidwe a midzi

1. How does communities members differenciate between research and relief project?

Kodi anthu amudzi mwanu amakusiyanitsa bwanji pakati pakafukufuku ndi pologilamu za mthandizi?

1. **Understanding of Randomisation studies and study procedures**
2. How would you describe community members understanding of the randomisation process? [probe: the randomisation process of the current study]

Kodi mungafotokoze bwanji mmene anthu ammudzi amamvetsera za ndondomeko yosankha mwa mwayi? [ fufuzani ndondomeko za kafukufuku amene akuchitika mudzi]

1. Do you know of any issues with the randomisation process?

Muli ndi maganizo ena alionse okhudza ndondomeko za wamwayi kapena masankhidwe anathu otenga nawo mbali?

1. **Myths and Misconceptions about the study?**
2. What myths exist about research? If there is myth, what factors contributed to it? How can understanding be promoted? How do we address the misconceptions?

Mukudziwapo za nkhani zilizonse, malingaliro, zikhulupiliro zokhuza kafukufuku, ngati zilipo tingatani kuti tithane nazo?

- - Prompt: If there any issues with the ongoing project and seek solutions for the mentioned problems?
  - Funsitsitsani: Ngati pali nkhani zokhuzana ndi pulojekiti yomwe ikuchitika ndipo pezani njira zothetsera mavuto omwe atchulidwa?

1. **Knowlegde about Informed Consent Process**
2. What communication strategies should be used to ensure comprehension of study information?

Kodi ndi njira ziti zolumikizirana zomwe zingagwiritsidwe ntchito kuti pakhale kumvetsetsa zofunikira za kafukufuku?

1. Kindly share us the informed consent procedures/processes which have been implemenetd with the project and any issues currently within the communitites?

Chonde tigaireni njira zomwe mumatengera chilolezo/ndondomeko zomwe zaikidwa ndi pulojekiti ndi zina zonse zomwe zikuchitika pano mmidzi?

1. What is the most good informed approach that the people of this community has preferred/would prefer.

Kodi ndi njira iti yabwino yomwe anthu a mmudzi uno amakonda/angakonde.

1. What is the best approach to provide information to people in this community? [ Probe: About verbal or signing consent, group, Group or individual]

Kodi ndi njira iti yabwino yopelekera uthenga kwa anthu mmudzi muno? [ Fufuzani za Kulankhula kapena kulemba, Pa gulu kapena payekha payekha]

1. **Information Sharing ( informational needs)**
2. What is the best approach to provide information to people in this community? [ probe about the verbal or written consent, group or individual]

Kodi ndi njira iti yabwino yopelekera uthenga kwa anthu mmudzi muno? [fufuzani: kulemba kapena kalata zapakamwa, pagulu kapena aliyense payenkha payenkha]

1. What is your experience in understanding the research information? [ probe about understanding of study risks, benefits, procedures, blood donation, dietary assessment needs, use of project bowls]

Munakumana ndi zotani kuti mumvetse nkhani yokhuza kafukufuku? [fufuzani zokhudza kuopsa kwa kafukufuku, phindu la kafukufuku, kupeleka magazi, kugawana chakudya, kugwiritsa ntchito mbale za kafukufuku, Kudya ufa, olemba zokhuzana ndi zakudya]

1. **Information needs/knoweldge gaps**
2. What information do people from this community need at this particular stage of th study? [ probe: about concerns or information gaps]
   1. Kodi anthu a Muudzi muno ali ndi uthenga wapadera womwe akufuna atamva kuchoera kwa ochita kafukufuku? [Fufuzani za madandaulo omwe alipo okhadza zochitza mukafukufuku]
3. How do people within the communities answer questions from other members of the community regarding the study? [ link response to concerns identified from the group about the overall study or the myths and misconceptions identified

Mukuganiza kuti anthu ammudzi angathe kuyankha mafunso kuchokera kwa anthu ena a mmudzi zokhuza kafukufukuyu?

1. Why do you think people do not have this information? [ probe about communication skills, settings of study in providing information]

Kodi mukuo ngati ndichifukwa chani anthu alibe mauthenga amenewa? [fufuzani za mapereekdwe kauthenga, ndondomeko zoperekera uthenga]

1. **Communiction Strategies**
2. What strategies were used to inform communities about the current research? [ Probe: Before enrollment community activties, field trip to bunda, flour proceessing trip]

Kodi annthu opanga kafukufuku akugwiritsa ntchito njira ziti popereka uthenga kwa anthu mudzi muno?

Fufuzani za misonkhano yodziwitsa anthu

Ulendo opita ku Bunda ku Lilongwe

Ulendo okakonza ufa ku Bunda

1. How do people perceive the communication strategies that were/are being used to inform people in this community anbout this research?

Kodi anthu amudzi muno akuti chani pazaubwino kapena kuipa kwa njira zoperekera mauthenga akafukfuku mudzi mwanu muno?

1. Have you encountered any issues in sharing information or answering questions from other members of the community?

Munayamba mwakumanako ndi zinthu zina popeleka uthenga kapena kuyankha mafunso kuchokera kwa anthu ena a mmudzi?

1. What strategies were used to inform communities about the current research? [ Probe: Before enrollment community activties, field trip to bunda, flour proceessing trip]
2. How (are) do community members share new information?

Anthu a mmudzi amagawa bwanji uthenga?

1. **Voluntariness:**
2. What do you understand about community members ability to participate in the study freely without being coehision?

Kodi mumamvetsa bwanji zokhuza anthu ammudzi kutengapo mbali mu kafukufuku mwa ufulu osakakamizidwa?

- 1. Probe about study participate ability to decide without fear
  2. Fufuzani za anthu otenga mbali kupanga chiganizo opanda mantha
  3. Especially the experience of scool going children and women
  4. Makamaka zomwe amakumana nazo ana opita ku sukulu ndi azimayi

1. How do community members perceive participating in research voluntarily?

Kodi anthu ganizo likuti kutenga mbali mukafukufuku ndikozipereka mosakakamizidwa amaliona bwanji?

1. what factors may force people to pa

Panthawiyi, muli ndi zoti mukufuna zinthu zasopano kuchokera kwa anthu opanga kafukufuku?

1. **Factors that influence Desicion Making Process within the communities**
2. How do people make decisions to participate in research in a houshold level?

Kodi antu apabanja amapanga bwanji chiganizo cotenga nawo mbali mukafukufuku?

1. What do you perceive are others peoples opinion significant in decision making in this community? [Probe:What roles does neighbors, heads of families, family members and members of the community have in the decision-making process?]

Ndi udindo wanji omwe anzanu, atsogoleri a mabanja, anthu a pabanja ndi anthu a mmudzi ali nawo popanga chisankho?

- - Fufuzani, zokhuza zomwe amakumana nazo ndi anthu a ma udindo, anthu omwe amakhala nawo

1. What impact does people with influence have on those who are participating in research? [ Probe: ability to withdrawal especially for children, aherence to eating flour,
   - 1. Kodi ndi Mumaganiza zotani za anthu omwe amatenga nawo mbali omwe ali ndi ma udindo mmudzi mukafukufuku ameneyu? [Fufuzani mmene amakhudzira anthu otenga nawo mbali popanga chisankho mwa ufulu potenga mbali mu kafukufuku, kutsatira ndondomeko za kafukufuku kapena chiganizo chosiya kuteng anawo mbali
2. **Role and Responsibility in recruting potential study members**
3. What factors motivate people to refuse to participate in research? If there are some who refused, why did they decide not to participate?

Kodi ndizifukwa ziti zomwe anthu amakanira kulowa kafukufuku? Ngati alipo udzimo anakana chifukwa chani?

1. What do community members perceive as their responsibility during research projects? [ probe if other people within their communtities have role]

Kodi muona kuti nkhani yaudindo womwe umabwera ndi kafukufuku anthu am mudzi muno amaiona bwanji? [ fufuzani za madyedwe a ufa mokhulupirika]

1. Do you think people in this community may have problems in eatin the flour? What challenges may people have? And How can this challenge be resolved?

Kodi ndi mavuto ati amene anthu otenga nawo mbali akhoza kukhala nawo omwe angawalepheretse kudya ufa mwandondomeko? Kodi vuto limeneli tingathane nalo bwanji?

1. How do people with responsibility within your village regard as their role to influence particpants to adhere to study intervtion [ probe: adhrence to eating the flour as expected]
2. Mukuganiza kuti udindo wanu ungapangitse otenga nawo mbali kupanga mbali yawo mwachitsanzo kudya ufa mmene mukuyembekezera?

**12.Motivation for study participation**

1. What do you think motivate people of this community to join the study?

Mukaganiza kuti chinawapangitsa anthu a mmudzi uno kulowa kafukufuku ndi chani?

1. In decision making what values do community members uphold first?

Popanga chiganizo ndi mfundo zanji zomwe anthu a mmudzi uno amayamba alingalira?

**13.Trust**

1. What concerns do people have abou the current research project? Whom do you think community members would approach first if there is an issue with their study participation? [ probe why would people approach that person, their role]
   1. Kodi anthu a mmudzi muno ali ndi madando ena alionse okhudza kafukufukuyu? Mukuganiza kuti anthu ammudzi angayambe kufikira ndani patakhala nkhani yokhudza kutenga nawo mbali kwawo mu kafukufuku? [fufuzani: Ndichifukwa chani angafikire munthu ameneyo, ndipo ali ndi udindo wotani]
2. What relationships has the study team built with the research communtiies here?

Kodi ndi ubale wanji umene anthu opangitsa kafukufuku apanga ndi anthu omwe akupanga nawo kafukufuku mmidzi yakuno?

**15.Factors affecting recruitment of study participants**

1. Do you think there may be any issues that may affect recruitment/participation of community members to the study? [ probe: Nature of the study activties (blood sample donation, use of special bowls foreating, non sharing concept of flour, the flour processing procedures, the maize production procedres, maize distriution procedure).

Mukuwona ngati pangakhale zina zomwe zingakhudze kulemba/kutenga nawo mbali kwa anthu ammudzi mu kafukufuku? [o Ndondomezo zake zakafuku-fuku, Zochitika zamukafukufuku monga, kupreka magazi, kugwirita nthcito ziwiya zosakhala zathu, malimidwe a chimanga, kupangidwe ka ufa, Chikhalidwe cha midzi yome ikutenga nawo mbali monga mapexedwe a chuma, Nkani za kasiyanidwe kapakati ka mai komanso abambo, Opangiri opanga chiganizo cotenga nawo mbali, kutenga nawo mbali mukafukufuku amene anachitika mbuyomu]

1. What are your experiences on study recruitment and experiences by the research community about these study activties?

Kodi anthu omwe akutenga mbali akukumana ndi zotani kama kotenga nawo gawo mukafukufuku?

1. Whom do you think can bestly approach communities for recruitment into research studies?

Kodi mukuganiza kuti ndi ndani amene angafikire bwino anthu ammudzi kuti apeze anthu olowa mu ma kafukufuku?

**16. Everyday experiences during participation in research ( Individualisation: Culture, Beliefs, Traditions and personality (Personal Ability)**

1. What are some of the specific issues according to your values, have you experienced in your day to day life due to your involevement in this project? [ probe: issues with flour distribution, eating flour everday, blood doation issues, no food sharing issues]

Kodi ndi zinthu zina ziti malingana ndi zikhulupiliro zanu, zomwe mwakumana nazo mmoyo wanu wa tsiku ndi tsiku chifukwa chakutenga gawo mu pulojekiti imeneyi? [ Fufuzani za ndondomeko zoawia ufa, kudya ufa wakafukufuku tsiku ndi tsiku, kupereka magazi ndi ndondomeko zina za kafukufuka]

**17. Benefits:**

1. What are peoples expectations from this research project?

Kodi anthu a mmudzi muno akuembekzera zotani kuchokera kukafukufukuyu?

1. How do other people look at this research project? What are the fears and concerns of the current project

Kodi anthu ena amulandira bwanji kafukufufukuyu? Kodi ubwino kapena kuipa kwa kafukufuku uyu ndi chani pakati panu?

1. THIS IS THE END OF THE QUESTIONNAIRE:
2. REMEMBER TO THANK THE RESPONDENT FOR THEIR TIME.
