# Supplementary material for: Operationalising a real-time research ethics approach: supporting ethical mindfulness in agriculture-nutrition-health research in Malawi
Source: BMC Med Ethics. 2022 Jan 11;23:3. doi: 10.1186/s12910-021-00740-1 (PMC8748184; doi:10.1186/s12910-021-00740-1)
Supplement: Supplementary file 2 — Additional file 2. Focus Group Discussion Guide Final 2019 Participants Phase 2. [file 12910_2021_740_MOESM2_ESM.docx]

1. **Experience about theresearch**
2. Please tell us about your experience from the time the project started till now?

Tiuzeni za kutenga mbali yanu mukafukufuku ameneyi?

If Yes: Prompt about previous rumours, SAE experiences, Rumours at this stage, their expectations

Fufuzani: Za manongonongo omwe alipo pano, malingaliro awo pazochitika zakafukufuku, zotsatira atadya ufa.

How would you describe understanding of research by the community at this stage?

- - 1. Pakali pano kodi anthu kumvetsatsa kwa kafukufuku ameneyu ndikotani?

1. How has the research experience helped people in this community understand more about research?

Kodi kafukufuku ameneyi wathandiza bwanji anthu amudzi muno kumvetsatsa zolinga kapena ndondomeko za kafukufuku?

1. Why do you value most as you take part in research?

Popanga chiganizo ndi mfundo zanji zomwe anthu a mmudzi uno amayamba alingalira?

1. What do you think reseachers conducting this study should consider most at this stage of the trial?

Kodi pakali pano anthu opanga kafukufuku ameneyi alingalire chani?

1. **Myths and Misconceptions about the study?**
2. What positive and negative myths exist about the activity of eating the flour nowresearch? If there is myth, what factors contributed to it?Are myths still existing?
   1. Mukudziwapo za nkhani zilizonse, malingaliro, zikhulupiliro zokhuza ufa omwe ukudyedwa?kafukufuku, ngati zilipo kufikira pano vuto likhoza kukhala chani? Nanga inu mukuziona bwanji zimenezi? tingatani kuti tithane nazo?
   2. Prompt: If there any issues with the ongoing project and seek solutions for the mentioned problems?
   3. Funsitsitsani: Ngati pali nkhani zokhuzana ndi pulojekiti yomwe ikuchitika ndipo pezani njira zothetsera mavuto omwe atchulidwa?
3. **Roles and Responsibilities**?
4. Can you share us how the following activities helped communities members consume the flour without any problems?
   1. Mungatifotokozere kuti kodi zochitika monga ulendo wa ku bunda, chionetsero chiphika mudzi chinathandiza anthu a mudzi muno bwanji kuti alandire udindo wakudya ufa mosavuta?
   2. Probe:Community sensitisation: Bunda visit, dish sharing meeting?
5. Do you think there are any issues that may impact the eating of the study flour?
   - 1. Kodi mukuona ngati pali zina zomwe zingalepheretse anthu kudya ufa mwandondomeko?
     2. Probe about addiitional family members, sharing of food with others, less food in the house. ( Probe what were their course of action if they were faced with this issues)
     3. Fufuzani:za anthu onjezera pabanja, zakugawana chakudya ku maliro, kapena chakudya kuchepa.
6. What do you think motivate people to eat the flour?
   - 1. Kodi mukuona ngati anthu akutsata ndondomeko zamadyedwe a Ufa chifukwa chani?
7. Do you think community members may influence study participants roles and responsibilities?
   1. Kodi mukuona ngati atsogoleri a mdera lanu angalimbikitse anthu kutsata ndondomeko za kafukufuku ameneyi?
      1. Probe about adherence to study activties, any social issues which are circulating or may emerge from other village members?
      2. Probe about key village people like the village head man roles, extension workers role, health survillance assistan role or houseold heads?
      3. Fufuzani: za mafumu, ma HSA, Alangizi azaulimi kapena zochitika mudzi zomwe zingasokoneze anthu kudya ufa.
8. How do you think study participants have handled their roles in this research project and tell us more about remaining study activities?
   1. Tiuzeni mmene udindo wanu wotenga nawo mbali waendera? Komanso tiunikireni ndondomeko zomwe zatsala kuti mukwaniritse kafukufuku asanathe?
9. Has study participants/community members experienced any issues as they eat the study flour?
   1. Kodi anthu a mudzi akumanako ndi vuto lililonse chiyambireni kudya ufa umenewu?
      1. Probe? Have you approached anyone else?Why would they approach that person?
      2. Fufuzani. Alipo mwamufotokozera za vuto lanu? Ndichifukwa chani mwamufikira munthu ameneyo?
      3. Probe? Who did they approach and why that person?
      4. Were they assisted? And Satisfied?
10. How (are) community members share new information?
11. Kodi anthu akugawana bwanji mauthenga osiyana siyana okhudza kakufukufu a
12. **Orientation to learning and motivation: Empowerment in Decision Making**
    1. Do you think there may be any issues that may affect participation of community members to the study?

Kodi pali kena kalikonse komwe kangasokoneze anthu chiganizo chotsatsira ndondomeko zakutenga nawo mbali mukafukufuku.?

- - 1. Prompt: About information gaps/knowlegde they are lacking.
    2. Probe SAE?, rumours, doubt?
  1. Do you think that people within the communities can answer questions from other members of the community regarding the study?

Mukuganiza kuti anthu ammudzi angathe kuyankha mafunso kuchokera kwa anthu ena a mmudzi zokhuza kafukufukuyu?

- 1. Do you think there are issues that are not well understood or the community require clarification on at this point?
  2. Kodi pali uthenga wina uliwonse womwe anthu sakumvetsa kapena angafune kuti amvetsetse pakali pano?

| 1. **Burden Sharing and Randomisation**    1. What do you think about study participants different roles and responsibilities?   Tifotokozere maganizo anu okudza maudindo osiyana aanth otenga nawo mbali pamene ena akudya ufa with ena wina koma palibe akudziwa.  Probe about: Other donating blood others not:  Household flour monitoring activties  Fufuzani: zamalingaliro awo pomwe ena akupereka magazi ena ai, ena akudya ufa wina ena ai?   - 1. What are the expectations of those who have donated blood and why?   Kodi malingaliro anu ndiotani pamene ena akupereka magazi ena ai?   - 1. Do you think this research has posed specific issues not encountered in your day to day life?   Kodi kafukufuku ameneyu wabweretsa zotani pamoyo wanu watsiku ndi tsiku kapena pakati panu mudzi muno? |
| --- |
