# Supplementary material for: Operationalising a real-time research ethics approach: supporting ethical mindfulness in agriculture-nutrition-health research in Malawi
Source: BMC Med Ethics. 2022 Jan 11;23:3. doi: 10.1186/s12910-021-00740-1 (PMC8748184; doi:10.1186/s12910-021-00740-1)
Supplement: Supplementary file 3 — Additional file 3. In-Depth Interviews Key Informants Phase 2. [file 12910_2021_740_MOESM3_ESM.docx]

**Particpants Profile:**

1. Would you please tell me about yourself, including what you do for living?

Mungandiuze zokhuza inuyo, komanso zomwe mumachita pamoyo wanu wa tsiku ndi tsiku?

1. What do you consider important about your job/role within the community?

Ndichani chomwe mumachiwona kuti ndi chopambana pa ntchito yanu/udindo wanu m’mudzi?

**Experience about research**

1. Tell us more about your experience in this research?
2. Tiuzeni za kutenga mbali yanu mukafukufuku ameneyi?
   1. What are some of your expectations

Malingaliro anu ndi otani?

- 1. How have you overcome the study rumours?

Mwathana nawo bwanji manongonongo?

- 1. What was your experience in eating the study flour?

Tifotokereni za ufa omwe mumadya?

- 1. Have you had any adverse Events?

Mwapezako vuto lililonse pamene mumadya ufa umenewu, ngai eya tiuzeni kuti munatani ndipo munathandizika bwanji? Munakhutira ndi thandizo lomwe munalandira?

1. How have you handled your roles in this research project and tell us more about remaining study activities?

Tiuzeni mmene udindo wanu wotenga nawo mbali waendera? Komanso tiunikireni ndondomeko zomwe zatsala kuti mukwaniritse kafukufuku asanathe?

a) If Yes: Prompt about previous upcoming activties like next blood donation.,

- 1. The significance or his role

Kufunikira kwa udindo wake

- 1. His overall exprience with such a role

Chidziwitso chake chonse ndi udindo umenewu

- 1. or any concerns from this role at this point?

kapena nkhawa zina zochokera ku udindo omwe ali nawo mukafukufuku panopa

1. how prepared are you for the remaining study activties?

Ndiokonzeka bwanji kumalizitsa ndondomeko za kafuku fuku?.

**Burden and Adoption**

1. How would you describe your understanding of your involvement on behalf of the community members especially in having different roles than everyone else??

Kodi mungafotokoze bwanji mmene mukumvwera kuti udindo wanu ndi osiyana ndi ena onse a mudzi muno maka potenga nawo mbali mukafuku fuku ameneyi? What are some of the issues which concerned you in regards tot his role personally?]

Kodi pali zina zomwe zimakhudzani kamba ka ndondomeko za kafukufuku zomwe inu panokha munaziona?

**Myths and Misconceptions about the study?**

1. What positive and negative myths exist about the activity of eating the flour now? Are myths still existing?
2. If yes, what do you think is the problem now?

Mukudziwapo za nkhani zilizonse, malingaliro, zikhulupiliro zokhuza ufa omwe ukudyedwa?, ngati zilipo kufikira pano vuto likhoza kukhala chani? Nanga inu mukuziona bwanji zimenezi? o Prompt: If there any issues with the ongoing project and seek solutions for the mentioned problems?

o Funsitsitsani: Ngati pali nkhani zokhuzana ndi pulojekiti yomwe ikuchitika ndipo pezani njira zothetsera mavuto omwe atchulidwa?

**Information needs/knoweldge gaps**

1. Do you think there may be any information needs of community members to the study team ?

Mukuwona ngati pangakhale kufunika kwa uthenga wapadera kapena kwa otenga nawo mbali kwa anthu ammudzi zokhudza zochitika mukafukufuku?

- - - Probe about information gaps/knowlegde they are lacking.

Fufuzani zokhuza kusiyana kwa chidziwitso/chidziwitso chomwe akusowa

- - - Probe about research team communication skills

Fufuzani za luso lolankhula la opanga kafukufuku

- - - Probe about the influence of family, neigdbors, cheifs, HSA, Commuity comittee members, Agriculture counsellors

Fufuzani za mphamvu ya banja, nzako. Mafumu, HSA, ma membala a komiti ya mmudzi, alangizi a zaulimi.

- - - About the distribution of flour to everyhousehold?

Zokhuza kugawa kwa ufa pa khomo lililonse?

1. Do you think that people within the communities can answer questions from other members of the community regarding the study?

Mukuganiza kuti anthu ammudzi angathe kuyankha mafunso kuchokera kwa anthu ena a mmudzi zokhuza kafukufukuyu?

- - Do you think there are issues that are not well understood or the community require clarification on at this point?
  - Kodi pali uthenga wina uliwonse womwe anthu sakumvetsa kapena angafune kuti amvetsetse pakali pano?

**Role and Responsibility in recruting potential study members**

1. Do you know of any community members who have refused to continue with the study? Mukudziwapo anthu ena a mmudzi omwe asiya kutenga nawo mbali mu kafukufukuyu?
   1. Why did they withdrawal?

Analekelanji kutenga nawo mbali?

- 1. What were the reasons that made them made a decision not to continue with the the study

Ndi zifukwa zanji zomwe zinawapangitsa kuti asankhe kusalowa nawo kafukufuku.

1. What did you do about this issues? Do you think you have a role to make sure people do not withdrawal??

Munapangapo chani zokhuza nkhani imeneyi? Mukuona ngati muli ndi udindo owonetsetsa kuti aliyense akupiriliza kutenga nawo mbali kafukufukuyu?

**Motivation for study participation**

1. What do you think is still motivating of this community to continue with the study?

Mukaganiza kuti chinawapangitsa anthu a mmudzi uno kupitiliza kutenga nawo mbali kafukufuku ndi chani?

1. In decision making what values do community members uphold first?

Popanga chiganizo ndi mfundo zanji zomwe anthu a mmudzi uno amayamba alingalira?

**Trust**

1. Have you experience any issues since you started eating the flour?

Kodi mwakumanako ndi vuto lililonse chiyambireni kudya ufa umenewu?

a. Probe? Have you approached anyone else?Why would they approach that person?

Fufuzani. Alipo mwamufotokozera za vuto lanu? Ndichifukwa chani mwamufikira munthu ameneyo?

1. b. What relationships has the study team built with the research communtiies here?

Kodi ndi ubale wanji umene anthu opangitsa kafukufuku apanga ndi anthu omwe akupanga nawo kafukufuku mmidzi yakuno?

Kodi mukuona ngati zinthu izi zingakhudze kalembera kapena chiganizo cha anthu chofuna kutenga nawo mbali mukafukufuku?

- - Nature of the study

Everyday experiences during participation in research ( Individualisation: Culture, Beliefs, Traditions and personality (Personal Ability)

What are some of the specific issues that you have experinced, learnt from this research project? Kodi ndi zinthu zina ziti zomwe mwapunzirapo chifukwa chotenga nawo mbali mukafuku fuku ameneyi?

1. THIS IS THE END OF THE QUESTIONNAIRE:
2. REMEMBER TO THANK THE RESPONDENT FOR THEIR TIME.
