# Supplementary material for: Operationalising a real-time research ethics approach: supporting ethical mindfulness in agriculture-nutrition-health research in Malawi
Source: BMC Med Ethics. 2022 Jan 11;23:3. doi: 10.1186/s12910-021-00740-1 (PMC8748184; doi:10.1186/s12910-021-00740-1)
Supplement: Supplementary file 4 — Additional file 4. In-Depth Interviews Key Informants Phase 1. [file 12910_2021_740_MOESM4_ESM.docx]

1. Would you please tell me about yourself, including what you do for living?

Mungandiuze zokhuza inuyo, komanso zomwe mumachita pamoyo wanu?

- - Prompt: How long have you been working at the current role? What he enjoys about his role especially relationship with community memberss ?
  - Funsitsitsani: Mwakhala nthawi yaitali bwanji mukugwira ntchito pa udindo umenewu? Mumasangalala ndi chani pa udindo wanu makamaka zokhuzana ndi anthu ammudzi?

1. What do you consider important about your job/role within the community?

Ndichani chomwe mumachiwona kuti ndi chopambana pa ntchito yanu/udindo wanu m’mudzi?

**Knowledge and experience about research**

1. Have you participated in any research?

Munayamba mwatengapo mbali mu kafukufuku aliyense?

- 1. What was the desin of the study

Mapangidwe ake akafukufuku anali otani?

- 1. What were the study activties?

Zochitika za mu kafukuku zinali zotani?

- 1. What were the benefits of the study?

Phindu la kafukufuku amaneyo linali chani?

- 1. What made you quliafy to particpate in the study

Chinakuyeneretsani ndi chani kuti mutenge nawo gawo mu kafukufuku?

1. Have you ever had a role in any other research activties?

Munayamba mwakhalako ndi udindo mu zochitika za mu kafukufuku wina aliyense?

a) If Yes: Prompt about previous research types,

Ngati ndi choncho: Funsitsitsani zokhuza mitundu ya akafukufuku a m’mbuyomu,

- 1. his role or involvement

udindo wawo kapena kutenga nawo mbali

- 1. The significance or his role

Kufunikira kwa udindo wake

- 1. His overall exprience with such a role

Chidziwitso chake chonse ndi udindo umenewu

- 1. or any concerns from previous research teams/projects

kapena nkhawa zina zochokera ku ma gulu a kafukufuku/ ma pulojekiti ammbuyomu

1. Were there any issues with the study? And how were these resolved.

Panali nkhani zina zokhuza kafukufukuyo? Ndipo nkhani zimenezi zinathetsedwa bwanji.

**Understanding of present research**

1. How would you describe how the community understand this research project ?

Mungafotokoze bwanji za mmene anthu ammudzi akumvetsera za kafukufuku ameneyu?

- - What do they achieve at the end of the study?

Kodi amapeza chani kumapeto kwa kafukufukuyu?

- - What are the benefits of taking part in the research?

Kodi phindu lotenga nawo mbali mu kafukufukuyu ndi chani?

- - What are the study activities?

Mukafukufukuyu mumachitika chani?

- - What are some of the risks of taking part in the study?

Kodi ndi ziopsezo zina ziti zomwe zimapezeka potenga nawo mbali mu kafukufuku?

- - Why is there village selected?

Ndichifukwa chani mudziwu unasankhiwa

1. Does communities members difference between research and relief project?

Kodi anthu a mudzi muno akutha kusiyanitsa pakati pa kafukufuku ndi mapologiramu a relief?

Understanding of Randomisation studies and study procedures

1. How would you describe community members understanding of the randomisation process?

Kodi mungafotokoze bwanji mmene anthu ammudzi amamvetsera za ndondomeko yosankha mwa mwayi?

1. What are the randomization process for this study? [ Probe on study arms where some will eat flour with selenium while others not]

Kodi ndondomeko za wamwayi kapena wachina ndani za kafukufuku uno ndizotani?

1. Do you know of any issues with the randomisation process? [Fufuzani zokhudza ufa wosiyaa womwe anthu adzadye wina wa selenium wina zinc]

Muli ndi maganizo ena alionse okhudza ndondomeko za wamwayi kapena masankhidwe anathu otenga nawo mbali?

**Myths and Misconceptions about the study?**

1. What positive and negative myths exist about research? If there is myth, what factors contributed to it?
2. How can understanding be promoted? How do we address the misconceptions?

Mukudziwapo za nkhani zilizonse, malingaliro, zikhulupiliro zokhuza kafukufuku, ngati zilipo tingatani kuti tithane nazo?

o Prompt: If there any issues with the ongoing project and seek solutions for the mentioned problems?

o Funsitsitsani: Ngati pali nkhani zokhuzana ndi pulojekiti yomwe ikuchitika ndipo pezani njira zothetsera mavuto omwe atchulidwa?

Knowlegde about Informed Consent Process

1. What do you understand is your role in study?

Mumamva kuti udindo wanu mu kafukufukuyu ndi otani?

- - - Prompt: To desribe the expectations of his role by the research team and research participants?
    - Funsitsitsani: Afotokoze zomwe a gulu la kafukufuku amayembekezera pa udindo wake komanso otenga nawo mbali
    - Probe about ensuring informed consent of study participants has been achieved?
    - Fufuzani za kuonetsetsa kuti chivomerezo cha otenga nawo mbali chachitika?
  - What communication strategies should be used to ensure comprehension of study information?

Kodi ndi njira ziti zolumikizirana zomwe zingagwiritsidwe ntchito kuti pakhale kumvetsetsa zofunikira za kafukufuku?

- - Kindly share us the informed consent procedures/processes which have been implemenetd with the project and any issues currently within the communitites?

Chonde tigaireni njira zomwe mumatengera chilolezo/ndondomeko zomwe zaikidwa ndi pulojekiti ndi zina zonse zomwe zikuchitika pano mmidzi?

- - What is the most good informed approach that the people of this community has preferred/would prefer.

Kodi ndi njira iti yabwino yomwe anthu a mmudzi uno amakonda/angakonde.

- - What is the best approach to provide information to people in this community?

Kodi ndi njira iti yabwino yopelekera uthenga kwa anthu mmudzi muno?

- - - Verbal or written

Kulankhula kapena kulemba

- - - Group or individual?

Pa gulu kapena payekha payekha

**Information Sharing ( informational needs)**

1. What is the best approach to provide information to people in this community?

Kodi ndi njira iti yabwino yopelekera uthenga kwa anthu mmudzi muno?

- 1. Verbal or written

Kulankhula kapena kulemba

- 1. Group or individual

Pa gulu kapena payekha payekha

- 1. Probe about their role and why?

Fufuzani za udindo wawo komanso chifukwa chani?

1. How did you learn about this trial?

Munadziwa bwanji za kafukufukuyu?

- - Probe about sensitisation meeting

Fufuzani za misonkhano yodziwitsa anthu

- - Field visit to Bunda in Lilongwe

Ulendo opita ku Bunda ku Lilongwe

- - Flour processing visit to Bunda

Ulendo okakonza ufa ku Bunda

1. How did the study team share the information about the study?

Anthu a kafukufuku anapereka bwanji uthenga okhuza kafukufuku?

1. What is your experience in understanding the research information?

Munakumana ndi zotani kuti mumvetse nkhani yokhuza kafukufuku?

- - Probe about study risks

Fufuzani zokhuza kuopsa kwa kafukufuku

- - Study benefits

Phindu la kafukufuku

- - Blood donation

Kupeleka magazi

- - Non food sharing activties

Zochitika zosakhudzana ndi kugawana chakudya

- - Use of study bowls

Kugwiritsa ntchito mbale za kafukufuku

- - The flour consumption

Kudya ufa

- - Dietary assessments

Zolemba zokhuzana ndi zakudya

**Information needs/knoweldge gaps**

1. Do you think there may be any information needs of community members to the study team ?

Mukuwona ngati pangakhale kufunika kwa uthenga wapadera kapena kwa otenga nawo mbali kwa anthu ammudzi zokhudza zochitika mukafukufuku?

- - - Probe about information gaps/knowlegde they are lacking.

Fufuzani zokhuza kusiyana kwa chidziwitso/chidziwitso chomwe akusowa

- - - Probe about research team communication skills

Fufuzani za luso lolankhula la opanga kafukufuku

- - - Probe about the influence of family, neigdbors, cheifs, HSA, Commuity comittee members, Agriculture counsellors

Fufuzani za mphamvu ya banja, nzako. Mafumu, HSA, ma membala a komiti ya mmudzi, alangizi a zaulimi.

- - - About the distribution of flour to everyhousehold?

Zokhuza kugawa kwa ufa pa khomo lililonse?

1. Do you think that people within the communities can answer questions from other members of the community regarding the study?

Mukuganiza kuti anthu ammudzi angathe kuyankha mafunso kuchokera kwa anthu ena a mmudzi zokhuza kafukufukuyu?

**Communiction Strategies**

1. What are some of the communications strategies that were used to help the communities understand study information?

Kodi ndi ndondomeko zina ziti zolumikizirana zomwe zinagwiritsidwa ntchito kuthandiza anthu a mmudzi kumvetsa za kafukufuku?

1. Have you encountered any issues in sharing information or answering questions from other members of the community?

Munayamba mwakumanako ndi zinthu zina popeleka uthenga kapena kuyankha mafunso kuchokera kwa anthu ena a mmudzi?

1. What do you understand about community members ability to participate in the study freely without being coehision?

Kodi mumamvetsa bwanji zokhuza anthu ammudzi kutengapo mbali mu kafukufuku mwa ufulu osakakamizidwa?

- - Probe about study participate ability to decide without fear

Fufuzani za anthu otenga mbali kupanga chiganizo opanda mantha

- - Especially the experience of scool going children and women

Makamaka zomwe amakumana nazo ana opita ku sukulu ndi azimayi

1. How (are) do community members share new information?

Anthu a mmudzi amagawa bwanji uthenga?

1. At this stage, do you have any issues that you require new information from the study team?

Panthawiyi, muli ndi zoti mukufuna zinthu zasopano kuchokera kwa anthu opanga kafukufuku?

Factors that influence Desicion Making Process within the communities

1. How would you explain decision-making norm in this community?

Mungalongosole bwanji mmene zisankho zimapangidwira mmudzimu?

1. What roles do neighbors, heads of families, family members and members of the community have in the decision-making process?

Ndi udindo wanji omwe anzanu, atsogoleri a mabanja, anthu a pabanja ndi anthu a mmudzi ali nawo popanga chisankho?

• Probe

Fufuzani

1. about thier experiences with people of influence, their social relationships

zokhuza zomwe amakumana nazo ndi anthu a ma udindo, anthu omwe amakhala nawo

1. If they play role, how are they involved? Prompt: E.g. discussions with them before approaching individuals. How?

Akatengapo mbali, amakhuzidwa motani? Funsitsitsani: E.g. kukambiranana nawo asanakumane ndi munthu payekhapayekha. Motani?

1. What do you think about the involvement of people with influence in your community in this study?

Mumaganiza zotani za anthu omwe amatenga nawo mbali omwe ali ndi ma udindo mmudzi mukafukufuku ameneyu?

1. Probe about their influence towards stuudy participants free will to participate in research

Fufuzani zokhuza mmene amakhudzira anthu otenga nawo mbali popanga chisankho mwa ufulu potenga mbali mu kafukufuku

1. adherence to eating flour?

Kutsatira kudya ufa?

1. Participants abiity to withdrawal from the study?

Kukwanitsa kutuluka mu kafukufuku?

Role and Responsibility in recruting potential study members

1. Do you know of any community members who have refused to enrol into this study?

Mukudziwapo anthu ena a mmudzi omwe anakana kulowa nawo mu kafukufukuyu?

- 1. Why did they refue

Anakaniranji

- 1. What were the reasons that made them made a decision not to join the study

Ndi zifukwa zanji zomwe zinawapangitsa kuti asankhe kusalowa nawo kafukufuku.

1. What did you do about this issues? Do you think you have a role to make sure eveyone joins the study?

Munapangapo chani zokhuza nkhani imeneyi? Mukuona ngati muli ndi udindo owonetsetsa kuti aliyense walowa kafukufukuyu?

**Motivation for study participation**

1. What do you think motivated people of this community to join the study?

Mukaganiza kuti chinawapangitsa anthu a mmudzi uno kulowa kafukufuku ndi chani?

1. In decision making what values do community members uphold first?

Popanga chiganizo ndi mfundo zanji zomwe anthu a mmudzi uno amayamba alingalira?

Trust

1. Whom do you think community members would approach first if there is an issue with their study participation?

Mukuganiza kuti anthu ammudzi angayambe kufikira ndani patakhala nkhani yokhudza kutenga nawo mbali kwawo mu kafukufuku?

a. Probe? Why would they approach that person?

Fufuzani. Ndichifukwa chani angafikire munthu ameneyo?

b. What is their role within the communities?

Udindo wawo ndiwotani mmidzi?

1. What relationships has the study team built with the research communtiies here?

Kodi ndi ubale wanji umene anthu opangitsa kafukufuku apanga ndi anthu omwe akupanga nawo kafukufuku mmidzi yakuno?

**Factors affecting recruitment of study participants**

1. Whom do you think can bestly approach communities for recruitment into research studies?

Kodi mukuganiza kuti ndi ndani amene angafikire bwino anthu ammudzi kuti apeze anthu olowa mu ma kafukufuku?

1. Do you think there may be any issues that may affect recruitment/participation of community members to the study?

Mukuwona ngati pangakhale zina zomwe zingakhudze kulemba/kutenga nawo mbali kwa anthu ammudzi mu kafukufuku?

1. What are your experiences on study recruitment and experiences by the research community about these study activties?

Mumakumana ndi zotani polowetsa anthu mu kafukufuku komanso anthu otenga nawo gawo mukafukufuku amakumana ndi zotani zokhuza zochitika za kafukufuku?

1. Do you think the following issues could affect participant recruitment?

Kodi mukuona ngati zinthu izi zingakhudze kalembera kapena chiganizo cha anthu chofuna kutenga nawo mbali mukafukufuku?

- - Nature of the study
  - Ndondomezo zake zakafuku-fuku?
  - Nature of the study activties (blood sample donation, use of special bowls foreating, non sharing concept of flour, the flour processing procedures, the maize production procedres, maize distriution procedure).
  - Zochitika zamukafukufuku monga, kupreka magazi, kugwirita nthcito ziwiya zosakhala zathu, malimidwe a chimanga, kupangidwe ka ufa)
  - Nature of the community (rural, economic status)
  - Chikhalidwe cha midzi yome ikutenga nawo mbali monga mapexedwe a chuma,
  - Gender
  - Nkani za kasiyanidwe kapakati ka mai komanso abambo
  - Decision-making norm (individual versus family versus community)
  - Opangiri opanga chiganizo cotenga nawo mbali
  - Previous exposure to research
  - Kutenga nawo mbali mukafukufuku amene anachitika mbuyomu?

1. What other factors affect participant recruitment?

Zilipo zina zomwe zingasokoneze chiganizo chotenga nawo mbali mukafukufuku?

Everyday experiences during participation in research ( Individualisation: Culture, Beliefs, Traditions and personality (Personal Ability)

1. What are some of the specific issues according to your values, have you experienced in your day to da life due to your involevement in this project?

Kodi ndi zinthu zina ziti malingana ndi zikhulupiliro zanu, zomwe mwakumana nazo mmoyo wanu wa tsiku ndi tsiku chifukwa chakutenga gawo mu pulojekiti imeneyi?

- - Probe for experiences for other family members at large, the research society at large

Fufuzani zomwe akumana nazo ena a pabanja, anthu ena otenga nawo kafukufuku

- - Probe against the following study procedures:

Fufuzani zokhuza ndondomeko zotsatirazi:

| 1. Flour production procedures?   Njira zokonzera ufa? | |
| --- | --- |
| 1. Flour distribution procedures?   Njira zogawira ufa? | |
| 1. Eating study flour every day?   Kudya ufa wa kafukufuku tsiku lililonse? |  |
| 1. My beliefs about sharing food?   Zikhulupiliro pankhani yogawana chakudya? |  |
| 1. Eating requirements ( to eat in separate plates as a family member)   Zoyenera pakudya ( kudya mu mbale yake yake monga munthu wa pa banja) |  |
| 1. Blood donation procedures?   Njira zopelekera magazi? |  |
| 1. My beliefs about blood donation or sample sharing with strangers?   Zikhulupiliro zanga zokhuza kupeleka magazi kapena kugawira magazi a chitsanzo kwa anthu osawadziwa? |  |
| 1. Dietary assessment visits?   Kuyendera za zotsatira za zakudya? |  |
| 1. Monitoring of the households to verify if we are eating the flour   Kuyang’anira ma nyumba kuwonetsetsa kuti tikudya ufa   1. The use of cards per household for flour ditribution?   Kugwiritsa ntchito ma card pa nyumba iliyonse kuti agawe ufa? |  |
| 1. Rumours about the study?   Manong’onong’o okhuza kafukufuku? |  |
| 1. Anxiety of family members and friends?   Nkhawa za anthu a pa banja ndi anzanu? |  |
| 1. Lack of freedom to decide whether to continue with the study due to my role (role as Villager, Household Head, Spouse, Minor)   Kupanda ufulu opanga chisankho kuti ndipitilize kupanga nawo kafukufuku chifukwa cha udindo wanga (udindo ngati wa mmudzi, mutu wa banja, mkazi/mamuna wa munthu, wachichepere) |  |

1. THIS IS THE END OF THE QUESTIONNAIRE:
2. REMEMBER TO THANK THE RESPONDENT FOR THEIR TIME.
