# Supplementary material for: Operationalising a real-time research ethics approach: supporting ethical mindfulness in agriculture-nutrition-health research in Malawi
Source: BMC Med Ethics. 2022 Jan 11;23:3. doi: 10.1186/s12910-021-00740-1 (PMC8748184; doi:10.1186/s12910-021-00740-1)
Supplement: Supplementary file 5 — Additional file 5. 3 RATS Checklist Limbanazo Matandika Nov 2020. [file 12910_2021_740_MOESM5_ESM.docx]

| **ASK THIS OF THE MANUSCRIPT** | **THIS SHOULD BE INCLUDED IN THE MANUSCRIPT** | **REFERENCE IN MANUSCRIPT** |
| --- | --- | --- |
| **R Relevance of study question** |  |  |
| Is the research question interesting? | Research question explicitly stated | The research question is explicitly stated towards the end of the background section pg 6 . |
| Is the research question relevant to clinical practice, public health, or policy? | Research question justified and linked to the existing knowledge base (empirical research, theory, policy) | The background section has shown what is existing in the area of study and has justified the research question (pgs.5-6). |
| **A Appropriateness of qualitative method** |  |  |
| Is qualitative methodology the best approach for the study aims?   - *Interviews:* experience, perceptions, behaviour, practice, process - *Focus groups:* group dynamics, convenience, non-sensitive topics - *Ethnography:* culture, organizational behaviour, interaction - *Textual analysis:* documents, art, representations, conversations | Study design described and justified i.e., why was a particular method (e.g., interviews) chosen? | The study design and the rationale for using Focus Group Discussions, In depth interviews and Observations and trial reports is justified (pg 12-14) |
| **T Transparency of procedures** |  |  |
| *Sampling* |  |  |
| Are the participants selected the most appropriate to provide access to the type of knowledge sought by the study?  Is the sampling strategy appropriate? | Criteria for selecting the study sample justified and explained   - *theoretical:* based on preconceived or emergent theory - *purposive:* diversity of opinion - *volunteer:* feasibility, hard-to-reach groups | Seven villages that had gatekeeper permission, challenges and serious rumours being circulated were purposively selected and trial participants together with their partners were conveniently We purposively sampled all the trial activities and trial reports. The criteria and justification are on pages 12-14 |
| *Recruitment* |  |  |
| Was recruitment conducted using appropriate methods? | Details of how recruitment was conducted and by whom | The researcher recruited recruited trial participants for focus group discussions and In-Depth interviews in Kasungu. The Obersarvation were conducted in Kasungu and Lilongwe The researchers used the Trial Implementing Team used sensitisation meeting platform to recruit potential study participants. The trial sensitisation coordinator introduced the study before any data collection exercise. This approach enabled the researchers to widely clarify the main objectives of the ethics engagement initiative. To enhance comprehension, information sheets were shared and those who could not read were asked to bring a witness. Letter of support was sought from Kasungu District Council and gate keepers approval was sought from Traditional Authority Wimbe and Village Head Men at Village level. (pg 16) |
| *Is the sampling strategy appropriate?* |  |  |
| Could there be selection bias? | Details of who chose not to participate and why | We recorded no refusals. All participants were information rich sources hence selected purposively or convinient hence no room for bias |
| *Data collection* |  |  |
| Was collection of data systematic and comprehensive? | Method(s) outlined and examples given (e.g., interview questions) | The methods followed in the study are outlined on pages 9-14. The interview guides are appended as additional files |
| Are characteristics of the study group and setting clear? | Study group and setting clearly described | The study group and setting are clearly described on pages 9-12 |
| Why and when was data collection stopped, and is this reasonable? | End of data collection justified and described | The period for data collection is described on Pg9 |
| *Role of researchers* |  |  |
| Is the researcher(s) appropriate? How might they bias (good and bad) the conduct of the study and results? | Do the researchers occupy dual roles (clinician and researcher)? Are the ethics of this discussed? Do the researcher(s) critically examine their own influence on the formulation of the research question, data collection, and interpretation? | The researchers had no dual roles. The ethical considerations in the study are described on page 15-16 The interpretation of the results is backed by participants quotes in the results section ( pgs 20 to 29. ) |
| *Ethics* |  |  |
| Was informed consent sought and granted? | Informed consent process explicitly and clearly detailed | Informed consent procedures described under the Methods section on page 8. |
| Were participants’ anonymity and confidentiality ensured? | Anonymity and confidentiality discussed | All quotes were anonymised and no research institution has been mentioned..(see pages 23 to 28 ) on Data management section |
| Was approval from an appropriate ethics committee received? | Ethics approval cited | The study was approved by the Colleege of Medicine Research Ethics Committee. See reference number on page 16. |
| **S Soundness of interpretive approach** |  |  |
| *Analysis* |  |  |
| Is the type of analysis appropriate for the type of study?   - *thematic:* exploratory, descriptive, hypothesis generating - *framework:* e.g., policy - *constant comparison/grounded theory:* theory generating, analytical | Analytic approach described in depth and justified  *Indicators of quality:* Description of how themes were derived from the data (inductive or deductive)  Evidence of alternative explanations being sought  Analysis and presentation of negative or deviant cases | The data analysis methods used is very suitable for the study and reference for the same has been mentioned on Page 14 |
| *Are the interpretations clearly presented and adequately supported by the evidence?* |  |  |
| Are quotes used and are these appropriate and effective? | Description of the basis on which quotes were chosen  Semi-quantification when appropriate  Illumination of context and/or meaning, richly detailed | Qoutes were chosen based on the rich information a quotation will provide. All quotes have been highlighted from page 9 to pages 21,. |
| Was trustworthiness/reliability of the data and interpretations checked? | Method of reliability check described and justified e.g., was an audit trail, triangulation, or member checking employed? Did an independent analyst review data and contest themes? How were disagreements resolved? | We used a coupled of data source methods that allowed u reach triangulation (see page 7 ) Additionally the reseachers shared their various code book and agreed on the emerging codes by consensus. To validate all codes, the codes were valiadated by the study collaborators see Page 8 and the code book sent as an additional file |
| *Discussion and presentation* |  |  |
| Are findings sufficiently grounded in a theoretical or conceptual framework?  Is adequate account taken of previous knowledge and how the findings add? | Findings presented with reference to existing theoretical and empirical literature, and how they contribute | Pages 9 .to 21 highlights the finds of the study. The findings also highlight previous findings from studies in different context discussing the same area of concern. … |
| Are the limitations thoughtfully considered? | Strengths and limitations explicitly described and discussed | We have cosnidred all aspect that could have challenged the implementation fo the study. See procedures to offset rattrition rate son page 21 |
| Is the manuscript well written and accessible? | Evidence of following guidelines (format, word count)  Detail of methods or additional quotes contained in appendix  Written for a health sciences audience | The RATS checklist is included as additional file |
| Are red flags present? These are common features of ill-conceived or poorly executed qualitative studies, are a cause for concern, and must be viewed critically. They might be fatal flaws, or they may result from lack of detail or clarity. | *Grounded theory:* not a simple content analysis but a complex, sociological, theory generating approach  *Jargon:* descriptions that are trite, pat or jargon filled should be viewed sceptically  *Over interpretation:* interpretation must be grounded in "accounts" and semi-quantified if possible or appropriate  *Seems anecdotal, self evident:* may be a superficial analysis, not rooted in conceptual framework or linked to previous knowledge, and lacking depth  *Consent process thinly discussed:* may not have met ethics requirements  *Doctor-researcher:* consider the ethical implications for patients and the bias in data collection and interpretation | None |

*The RATS guidelines modified for BioMed Central are copyright Jocalyn Clark. They can be found in Clark JP:*How to peer review a qualitative manuscript*. In*Peer Review in Health Sciences*. Second edition. Edited by Godlee F, Jefferson T. London: BMJ Books; 2003:219-235*
