# Supplementary material for: Operationalising a real-time research ethics approach: supporting ethical mindfulness in agriculture-nutrition-health research in Malawi
Source: BMC Med Ethics. 2022 Jan 11;23:3. doi: 10.1186/s12910-021-00740-1 (PMC8748184; doi:10.1186/s12910-021-00740-1)
Supplement: Supplementary file 6 — Additional file 6. Self-Efficacy Assessment Tools for Study Participants 2019. [file 12910_2021_740_MOESM6_ESM.docx]

**INDIVIDUAL SELF-EFFICACY ASSESSMENT SCORE CARD FOR STUDY PARTICIPANTS**

| **1.Focused Interactions Communication (Informed Consent)** | | | | | |
| --- | --- | --- | --- | --- | --- |
|  | Very bad | bad | Good | Very good | Excellent |
| The information shared by the research team is sufficient to help me answer questions asked by community members |  |  |  |  |  |
| The information sharing sessions allowed me to express myself freely? |  |  |  |  |  |
| My indirect messages were effectively addressed |  |  |  |  |  |
| My indirectly expressed needs were discussed |  |  |  |  |  |
| My concerns and fears indirectly expressed were dealt with (Indirect-implied by verbal/non-verbal expressions) |  |  |  |  |  |
| My concerns and fears directly expressed were dealt with (Direct-explicitly verbally stated) |  |  |  |  |  |
| My concerns/fears wear dealt with facts |  |  |  |  |  |
| I was given time to interact with the research team |  |  |  |  |  |

This section will allow the study team assess the capability of the study participant’s ability to offset myth and misconceptions about the study, to report serious adverse events, to ask and share information with others. This assessment tool will allow the study assess the self-efficacy of study participants.

| **2.Verbal Persuasion: Self-Evaluation (Personal Ability)** | | | |
| --- | --- | --- | --- |
|  | Not at all True | Hardly True | Exactly True |
| I am confident that I could deal efficiently with myths and misconceptions of the study |  |  |  |
| I am confident that I can ask questions to the study team |  |  |  |
| I am confident that I can report any serious adverse event to the study team representatives |  |  |  |
| I can share study information with other members of the community without problems |  |  |  |
| Once I have decided to participate in the study, I will try hard to comply to the requirements of the study |  |  |  |
| I am confident that I cannot be influenced by any myths and misconceptions of the study from the community members |  |  |  |
| I can easily dispute wrong information about the study and tell people about the correct information |  |  |  |
| I am confident that I cannot be influenced by the community members about my involvement in the study |  |  |  |
| I am confident that I can withdrawal from participating in the study if I wish at any time? |  |  |  |
| I am confident that I will complete my study activities |  |  |  |

| **3.Individualisation: Culture, Beliefs, Traditions and personality (Personal Ability)** | | | |
| --- | --- | --- | --- |
|  | Not at all True | Hardly True | Exactly True |
| •Do you think the following issues could affect participant adherence to study procedures or study recruitment? | | | |
| Eating study flour every day? |  |  |  |
| I am confident that I can report any serious adverse event to the study team representatives |  |  |  |
| Blood donation procedures? |  |  |  |
| Dietary assessment visits? |  |  |  |
| Rumours about the study? |  |  |  |
| Anxiety of family members and friends? |  |  |  |
